# A Skull Bone Marrow‐to‐Brain Axis Links Osteoblastic Activity to Myeloid Cell Trafficking, Cerebral Blood Flow, and Cognition in Alzheimer's Progression

**DOI:** 10.1002/advs.75622

**Published:** 2026-05-10

**Authors:** Lei Xiong, Dong Sun, Hao‐Han Guo, Daehoon Lee, Zhipeng Liu, Lin Mei, Wen‐Cheng Xiong

**Affiliations:** ^1^ Department of Neurosciences School of Medicine Case Western Reserve University Cleveland Ohio USA; ^2^ Chinese Institute of Medical Research Capital Medical University Beijing China

**Keywords:** Alzheimer's disease, APP, ATP6AP2, dural channels, meninges, osteoblasts, skull bone marrow

## Abstract

Patients with Alzheimer's disease (AD) often develop osteoporosis, but the role of bone remodeling in AD remains unclear. We previously showed that osteoblast‐specific expression of APP_swe_ induces bone loss, glial activation, and behavioral deficits, suggesting a bone‐to‐brain signaling axis. Here, we identify an altered skull bone marrow (SBM)‐to‐brain axis in AD. Early SBM changes, including reduced cellularity, increased density, and expanded vascular channels to the meninges, occur in multiple APP_swe_ mouse models. These vascular changes facilitate migration of SBM‐derived myeloid cells into the meninges and cortex, improving cerebral blood flow (CBF) and slowing cognitive decline. Notably, these effects are age‐dependent, emerging at 6 months but diminishing by 12 months. Enhancing this axis via bone marrow transplantation improves CBF and cognitive function in aged mice, whereas disrupting it through osteoblastic deletion of ATP6AP2 impairs both. Together, these findings reveal a previously unrecognized SBM‐to‐brain axis that regulates immune, vascular, and cognitive functions, highlighting systemic contributions to AD pathogenesis.

## Graphical abstract

This study reveals that Alzheimer's disease–linked APP expression in bone‐forming cells drives skull bone marrow remodeling and alters its vascular connections to the brain. These changes disrupt immune cell trafficking, cerebral blood flow, and cognition. Targeting bone marrow macrophages restores brain function, highlighting a previously unrecognized bone–brain axis in Alzheimer's disease progression.

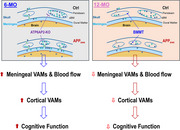

## 1 Introduction

Emerging evidence indicates that alterations in the communications between skull bones, meninges, and the brain may play important roles in the pathogenesis of various neurological diseases, including traumatic brain injury (TBI), stroke, multiple sclerosis (MS), subarachnoid hemorrhage, meningitis, brain tumor metastasis, and neurodegenerative diseases such as Alzheimer's disease (AD) and Parkinson's disease (PD) [1, 2, 3, 4]. Interestingly, AD often coincides with osteoporosis, another age‐associated chronic degenerative disease [5, 6, 7, 8, 9, 10, 11, 12]. Although traditionally viewed as separate disorders, AD and osteoporosis share several common factors, such as chronic inflammation, oxidative stress, cellular senescence, and hormonal deficiencies [13, 14]. However, the relationship between AD and osteoporosis, and whether AD genetic risk factors affect skull bone (SB), skull bone marrow (SBM) remodeling, and the connections linking SBM with the meninges, remains poorly understood.

The App gene is a well‐known Mendelian factor in early‐onset AD, with the detrimental effects of the Swedish mutant APP (APP_swe_) on AD pathogenesis extensively documented [15, 16, 17]. For instance, the *Tg2576* transgenic model, which expresses human APP_swe_ under the control of a prion promoter, and the newer APP mutant knock‐in (KI) mouse model, *APP^NL‐G‐F^*, both develop neuro‐pathological deficits that resemble aspects of AD in aged mice [18, 19]. While APP is widely expressed, including in osteoblasts (bone‐formation cells) and osteoclasts (bone‐resorbing cells) [20, 21], research on the role of APP and APP_swe_ in bone remains limited. Notably, both App knockout (*App^−/−^*) and *Tg2576* mice exhibit early osteoporotic‐like traits that appear before neuro‐pathological changes [20, 21, 22]. These findings underscore the involvement of APP and APP_swe_ in bone remodeling and homeostasis. However, it remains unclear whether these bone deficits impact brain function. Given that bone remodeling affects marrow in various types of bones, it is yet to be determined whether altered bone remodeling due to APP_swe_ influences the development and function of SBM and its vascular connections.

To address these questions, we initially generated *TgAPP_swe_^hOCN‐Cre(tg)^* mice by crossing *loxP‐STOP‐loxP (LSL)‐APP_swe_* with *hOCN‐Cre(tg)*, a transgenic line expressing Cre under the control of human osteocalcin (BGLAP) promoter (Ocn/Bglap) [21, 23]. These mice developed not only osteoporotic‐like bone deficits but also increased activation of glial cells selectively in the cortex, including GFAP^+^ reactive astrocytes and IBA1^+^ microglia/macrophages, inflammatory cytokines, and behavioral changes [23]. While these findings support a bone‐to‐cortex axis in AD development, we unexpectedly detected the Cre/APP_swe_’s leaky expression in select brain regions, including the dorsal dentate gyrus, olfactory bulb, and cerebellum in this mouse line. [23, 24] Although the neuronal expression was weaker than that in osteoblasts [23], it introduces potential confounding effects that may complicate interpretation of the brain and behavioral phenotypes observed in these mice.

In this study, we addressed the limitations of previous models by generating a new mouse line, *TgAPP_swe_^mOCN‐Cre^*, in which *LSL‐APP_swe_* were crossed with *mOCN‐Cre(KI)*, a newly generated Cre knock‐in mouse line that expresses Cre under the control of endogenous *Ocn/Bglap* promoter. This new mouse line specifically expressed APP_swe_/Cre in osteoblast‐lineage cells, without the “leaky” neuronal expression observed in *TgAPP_swe_^hOCN‐Cre(tg)^* mice. These newly generated mice faithfully recapitulated both the skeletal and cortical phenotypes seen in the previous model, further supporting the existence of a bone‐to‐cortex axis in AD progression.

Using this refined model, we investigated mechanisms underlying cortex vulnerability to osteoblastic APP_swe_ expression. Our results show that APP_swe_ expression in osteoblasts induces early structural changes in the SBM and associated channels, including reduced marrow volume, increased SBM density, and enhanced channels connecting SBM to the dural meninges. Notably, these anatomical changes were age‐dependent: most prominent at 6 months of age (6‐MO) but declined by 12 months (12‐MO), correlated well with alterations in meningeal vessel‐associated macrophages (VAMs), cortical microglia, cerebral blood flow (CBF), and cognitive performance. Importantly, similar SBM and meningeal phenotypes were also detected in other AD models, such as *Tg2576* and *APP^NL‐G‐F^* mice. Importantly, bone marrow macrophage transplantation (BMMT) into 12‐MO *TgAPP_swe_^mOCN‐Cre^* mice increased macrophage/microglia populations in the SBM, meninges, and cortex, and improved CBF and cognitive function. Further mechanistic insights from RNA‐seq analysis of APP_swe_^+^ calvarial osteoblasts revealed alterations in multiple pathways, including cell adhesion and proliferation and Wnt/β‐catenin pathways. Mice with osteoblastic depletion of ATP6AP2, a Wnt/β‐catenin signaling and v‐ATPase regulator implicated in dementia, showed disrupted SBM and channel formation, reduced meningeal and cortical macrophages/microglia, diminished CBF, and impaired cognitive function, in control and *TgAPP_swe_^mOCN‐Cre^* mice. In summary, these findings underscore the critical roles of osteoblastic APP_swe_ and ATP6AP2 in regulating the development and remodeling of the SBM and its channels. They also highlight a novel and important SBM–meninges–cortex signaling axis that contributes to the pathophysiology of AD.

## 2 Results

### 2.1 Earlier Onset Changes in SBM in *TgAPP_swe_^mOCN‐Cre^* Mice that Specifically Express APP_swe_ in Osteoblast‐Lineage Cells

To investigate a bone‐to‐brain axis in AD development, it is critical to establish a mouse line that specifically expresses AD risk gene in bone cells (e.g., osteoblasts) without leaky expression in the brain and other tissues. To this end, we generated a new knock‐in mouse line, mOCN‐Cre [also referred to as mOCN‐Cre(KI)], by inserting a P2A‐Cre cassette at the C‐terminus of the endogenous *Ocn/Bglap* gene using CRISPR/Cas9‐mediated genome editing (Figure S1A). This insertion did not disrupt endogenous *Ocn/Bglap* expression, as RT‐PCR analysis showed comparable *Bglap* transcript levels in bone marrow stromal cells (BMSCs) from wild‐type and mOCN‐Cre mice (Figure S1B).

To assess tissue/cell type specificity of the Cre expression, we crossed mOCN‐Cre mice with the Ai9 Cre‐responsive tdTomato reporter line to generate *mOCN‐Cre;Ai9* mice. In these animals, tdTomato expression was restricted to osteoblast‐lineage cells in the bone, with minimal to no expression detected in the brain (Figure S1C, left panel) or other tissues examined (data not shown). This contrasts with *hOCN‐Cre(tg)* mice, which exhibited tdTomato expression in discrete brain regions due to transgene leakiness (Figure S1C, right panel) [23, 24]. These results confirm that mOCN‐Cre drives osteoblast‐specific Cre activity, consistent with Ocn's established role as a marker of bone‐forming osteoblasts [25, 26].

We next crossed *mOCN‐Cre* mice with *LSL‐APP_swe_* to generate *TgAPP_swe_^mOCN‐Cre^* mice (Figure S1D). As expected, APP_swe_ expression in this new line was confined to osteoblast‐lineage cells, with no detectable expression in the cortex or hippocampus (Figure S1E).

**FIGURE 1:**
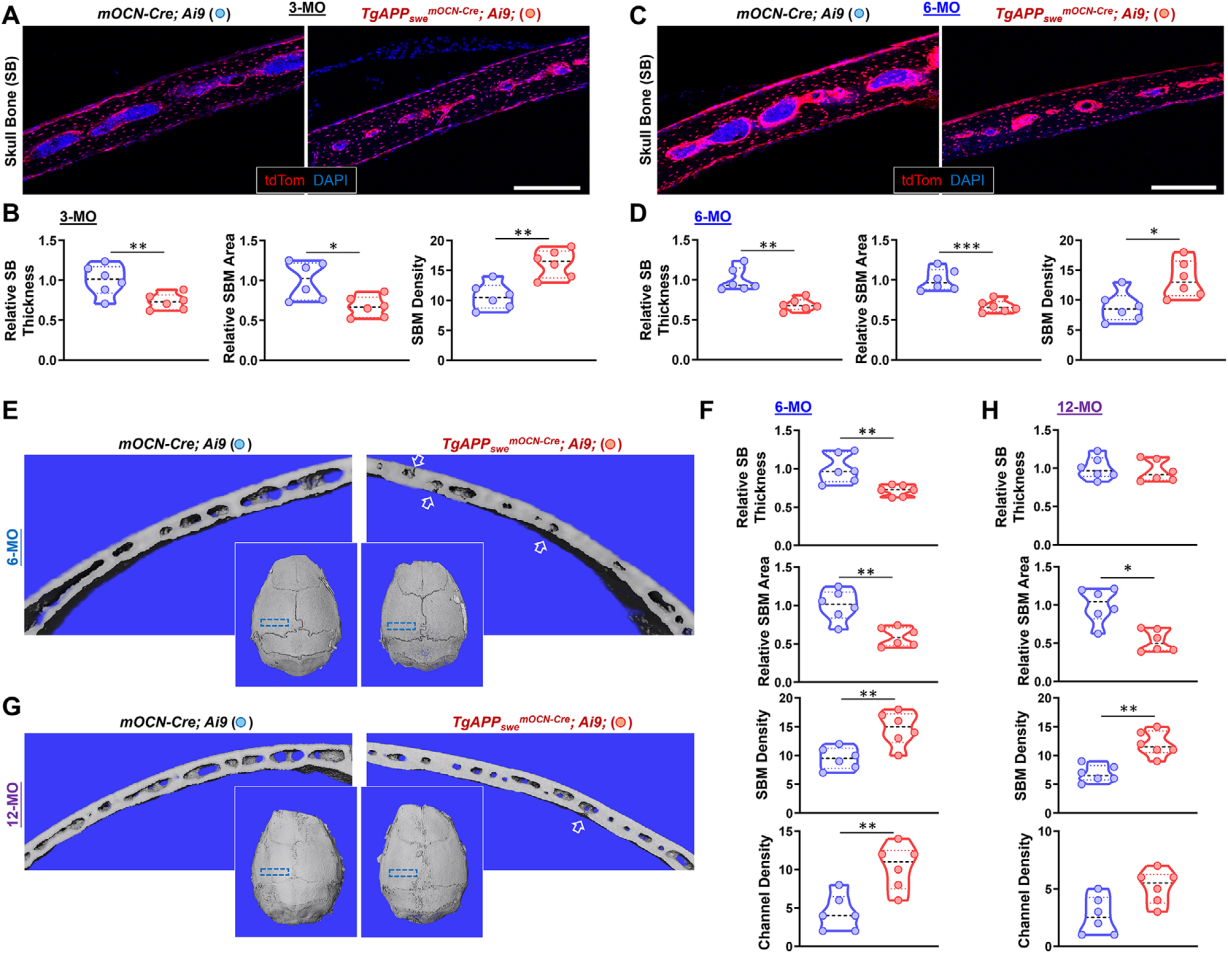
Earlier onset skull bone deficits in Tg*APP_swe_^mOCN‐Cre^* mice. (A) Representative images of skull bone sections from 3‐MO *mOCN‐Cre; Ai9* and *TgAPP_swe_^mOCN‐Cre^; Ai9* mice immunostained with DAPI (blue). Cre‐positive cells express the red fluorescent protein tdTomato (red). Scale bar, 0.5 mm. (B) Quantification analyses of skull bone (SB) thickness, skull bone marrow (SBM) area, and SBM density in A. (C) Representative images of skull bone sections from 6‐MO *mOCN‐Cre; Ai9* and *TgAPP_swe_^mOCN‐Cre^; Ai9* mice. Scale bar, 0.5 mm. (D) Quantification analyses in C. (E) Representative µCT 3D images of skull bone from 6‐month male ctrl and *TgAPP_swe_^mOCN‐Cre^* mice littermates. (F) Quantification analyses of SB thickness, SBM area, SBM density, and channel density by direct model of µCT analysis in E. (G) Representative µCT 3D images of skull bone from 12‐month male ctrl and *TgAPP_swe_^mOCN‐Cre^* mice littermates. (H) Quantification analyses of µCT analysis in G. Data in (B), (D), (F), and (H) are shown as violin plots together with individual data points, and dot lines and dash line represent the quartiles and the median, respectively (n  =  6 male mice of each genotype). P values obtained by unpaired two‐tailed *t*‐test. *P < 0.05. **P < 0.01. ***P < 0.001.

We then investigated the skeletal phenotypes in the femur, vertebrae, and skull by immunohistochemical analysis of bone sections from various ages of control (*mOCN‐Cre;Ai9*) and *TgAPP_swe_^mOCN‐Cre^;Ai9* mice. At 3‐MO, tdTomato‐based assessment revealed minimal tdTomato^+^ osteoblast bone volume (OBV) changes in the femur and vertebrae of mutants (Figure S1F,G). However, parietal calvarial sections from 3‐MO *TgAPP_swe_^mOCN‐Cre^;Ai9* skulls showed marked abnormalities compared to controls, including reduced skull bone (SB) thickness, decreased SBM volume, and increased SBM density (Figure 1A,B). By 6‐MO, these skull abnormalities persisted, and significant reductions in OBV/TV (total volume) were also observed in mutant femur and vertebrae (Figure 1C,D; Figure S1F,H).

We further validated the skull bone changes by microCT imaging analysis. Consistent with the immunohistological data, 6‐MO *TgAPP_swe_^mOCN‐Cre^* mice exhibited significantly thinner skull bones and more numerous, smaller SBM cavities relative to controls (Figure 1E,F).

We next examined whether these skull phenotypes persist with age. MicroCT analysis of 12‐MO *TgAPP_swe_^mOCN‐Cre^* mice, compared with age‐matched controls, revealed an increased number of smaller BM cavities (Figure 1G,H), similar to those observed in 6‐MO mutants. In contrast to the 6‐MO phenotype, however, SB thickness in 12‐MO mutants appeared to be comparable to those in control mice (Figure 1G,H).

Together, these results indicate that APP_swe_ reduces SB thickness and alters SBM remodeling and demonstrate that SB and SBM are particularly vulnerable to early pathological changes driven by osteoblastic APP_swe_ expression.

### 2.2 Elevated Vascular Channels Connecting SBM to the Dura Mater and Increased BM Cells in Dura Mater in 6‐MO, but not 12‐MO, *TgAPP_swe_^mOCN‐Cre^* Mice

**FIGURE 2:**
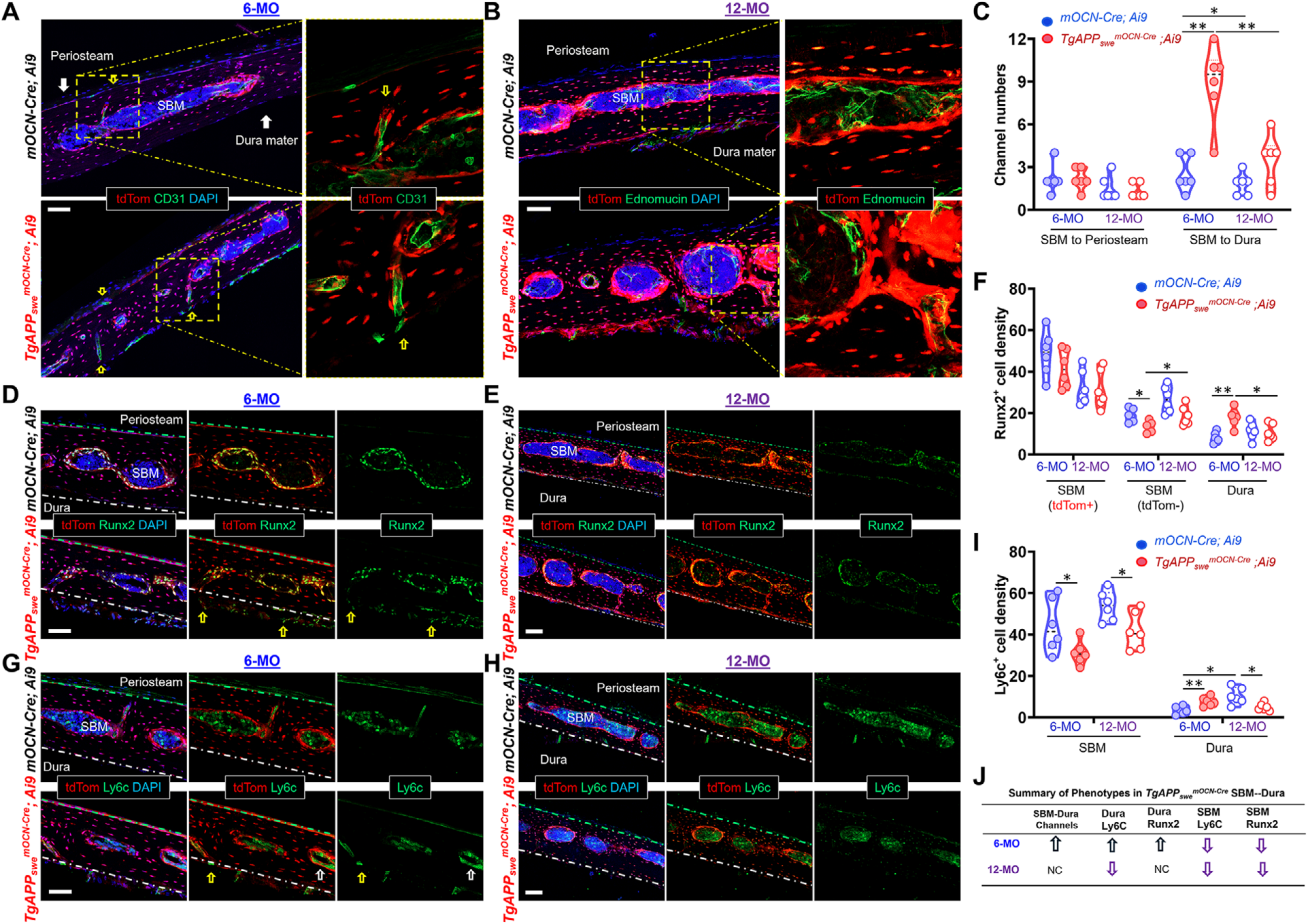
Increased vascular channels and dura side myeloid cell distribution in 6‐MO, but not 12‐MO, *TgAPP_swe_^mOCN‐Cre^* mice. (A,B) Representative images of skull bone sections from 6‐MO (A) or 12‐MO (B) male *mOCN‐Cre; Ai9* and *TgAPP_swe_^mOCN‐Cre^; Ai9* mice coimmunostained with CD31 (green) (A) / ednomucin (green) (B) and DAPI (blue). Cre‐positive cells express the tdTomato (red). Scale bar, 100 µm. (C) Quantification analyses of channel numbers from SBM to periosteam and dura in A‐B. (D,E) Representative images of skull bone sections from 6‐MO (D) or 12‐MO (e) male *mOCN‐Cre; Ai9* and *TgAPP_swe_^mOCN‐Cre^; Ai9* mice coimmunostained with Runx2 (green) and DAPI (blue). Cre‐positive cells express the tdTomato (red). Scale bar, 100 µm. (F) Quantification analyses of tdTom^+^Runx2^+^ and tdTom^−^Runx2^+^ cell density in SBM, and Runx2^+^ cell density in dura side in D‐E. (G,H) Representative images of skull bone sections from 6‐MO (G) or 12‐MO (H) *mOCN‐Cre; Ai9* and *TgAPP_swe_^mOCN‐Cre^; Ai9* mice coimmunostained with Ly6c (green) and DAPI (blue). Cre‐positive cells express the tdTomato (red). Scale bar, 100 µm. (I) Quantification analyses of Ly6c^+^ cell density in SBM and dura side in G‐H. (J) Summary of phenotypes in *TgAPP_swe_^mOCN‐Cre^* SBM‐Dura. Data in (C), (F), and (I) are shown as violin plots together with individual data points, and dot lines and dash line represent the quartiles and the median, respectively (*n*  =  6 male mice of each genotype). Statistical analysis was performed using two‐way ANOVA followed by Bonferroni's post hoc test. *P < 0.05. **P < 0.01.

Notably, in addition to the increased SBM density, microCT imaging revealed an expansion of channel‐like structures connecting the SBM to the dura mater in 6‐MO *TgAPP_swe_^mOCN‐Cre^* mice (Figure 1E,F). Given recent reports identifying bone‐embedded vascular channels that link the SBM directly to meningeal vasculature [1, 27, 28, 29, 30], we speculate that the microCT‐detected structures may represent such conduits. To test this, we performed co‐immunostaining on skull (parietal calvaria) sections from 6‐MO *TgAPP_swe_^mOCN‐Cre^;Ai9* and control (*mOCN‐Cre;Ai9*) mice using endothelial markers (CD31, Endomucin). As expected, vascular channels traversing the bone and connecting the SBM with the dura mater as well as the periosteum were identified (Figure 2A, open arrows). These channels were surrounded by tdTomato^+^Runx2^+^ osteoblasts, RANK^+^ osteoclasts, and IBA1^+^ myeloid cells (Figure S2A). Importantly, the number of channels connecting the SBM to the dura, but not to the periosteum, was significantly increased in 6‐MO *TgAPP_swe_^mOCN‐Cre^;Ai9* mice (Figure 2A,C), corroborating the microCT findings (Figure 1E,F). However, SBM‐to‐dura channel numbers in 12‐MO mutants appeared to be comparable to those in control mice (Figures 1G,H and 2B,C). These results imply that osteoblastic APP_swe_ expression promotes SBM‐to‐dura connectivity at 6‐MO age, indicating active remodeling of SBM and vascular architecture.

Prompted by this age‐dependent changes in SBM‐to‐dura connectivity in the mutant mice, we asked whether SBM‐derived cells exhibit altered migratory behavior toward their dura. Co‐immunostaining for mesenchymal stromal (Runx2), CAR (CXCL12), and myeloid (Ly6C, IBA1) cell markers were performed in 6‐ and 12‐MO mutant and control SB sections (Figure 2D–I; Figure S2B–G). At 6‐MO, while the total number of tdTomato^+^ cells (BMSCs, osteoblasts and osteocytes) remained largely unchanged, a reduction of Runx2^+^tdTom^−^ and CXCL12^+^tdTom^−^ stromal/CAR cells in the SBM, accompanied by an increase of these cells in the dura, were detected in *TgAPP_swe_^mOCN‐Cre^* mice (Figure 2D,F; Figure S2B,D). Similarly, Ly6C^+^ and IBA1^+^ myeloid cells were reduced in the SBM but enriched along vascular channels and in the dura in the 6‐MO mutants (Figure 2G,I; Figure S2E,G). Notably, these myeloid cells displayed an elongated morphology and close association with tdTom^+^ osteoblasts surrounding the SBM and channels in mutants, suggesting a migratory phenotype (Figure 2G; Figure S2A). By 12‐months, the numbers of RUNX2^+^tdTom^+^ osteoblasts in skull bone or surrounding the SBM, channels, and dura were all marked reduced or undetectable in both control and mutants (Figure 2E,F), concordant with the decline of channel abundance in both 12‐MO control and mutants. Additionally, the CXCL12^+^tdTom^−^ CAR cells in the SBM and dura remained lower in the mutants (Figure S2C,D); and the dural distributions of Ly6C^+^ and IBA1^+^ myeloid cells seen at 6‐MO were no longer evident in 12‐MO mutants (Figure 2H–J; Figure S2F,G).

Although the reduced SBM cells appeared to be linked with the increased SBM cells’ distribution in dura mater in 6‐MO mutant mice (Figure 2J), implicating an increased cell migration to underlie the altered cell distribution, it remains unclear whether osteoblastic APP_swe_ expression affects the proliferative capacity of SBM‐resident cells. To address this question, we examined proliferation within SBM macrophages by co‐immunostaining for IBA1 and Ki67. In 6‐MO *TgAPP_swe_^mOCN‐Cre^* mice, the IBA1^+^Ki67^+^ cell density and proportion of proliferating IBA1^+^Ki67^+^ cells among total IBA1^+^ cells were slightly, but not significantly, reduced compared with controls (Figure S3A–D). This reduction became more pronounced with age, as 12‐month‐old (12‐MO) mice exhibited a significant decline in the proliferative capacity of SBM IBA1^+^ cells relative to 6‐MO mice, with a further decrease observed in the mutant group (Figure S3A–D). These findings, together with the reduced number of SBM myeloid cells and the increased accumulation of SBM‐derived cells in the dura mater at 6‐MO, suggest that the decrease in SBM IBA1^+^ cells in 6‐MO mutants may reflect enhanced migration and a slight reduction of the cell proliferation, whereas in aged or 12‐MO mutants, impaired proliferation appears to be the predominant factor.

Together, these findings suggest an age‐dependent, cell non‐autonomous role of osteoblastic APP_swe_ in regulating SBM remodeling, vascular channel dynamics, and the distribution, morphology, and proliferation of marrow cells, particularly myeloid populations, highlighting a potential pathway through which SBM changes could influence brain function in AD.

### 2.3 Increased Meningeal Vascular‐Associated Macrophages, Cortical Microglia, and Cerebral Blood Flow in 6‐MO, but not 12‐MO, *TgAPP_swe_^mOCN‐Cre^* Mice

**FIGURE 3:**
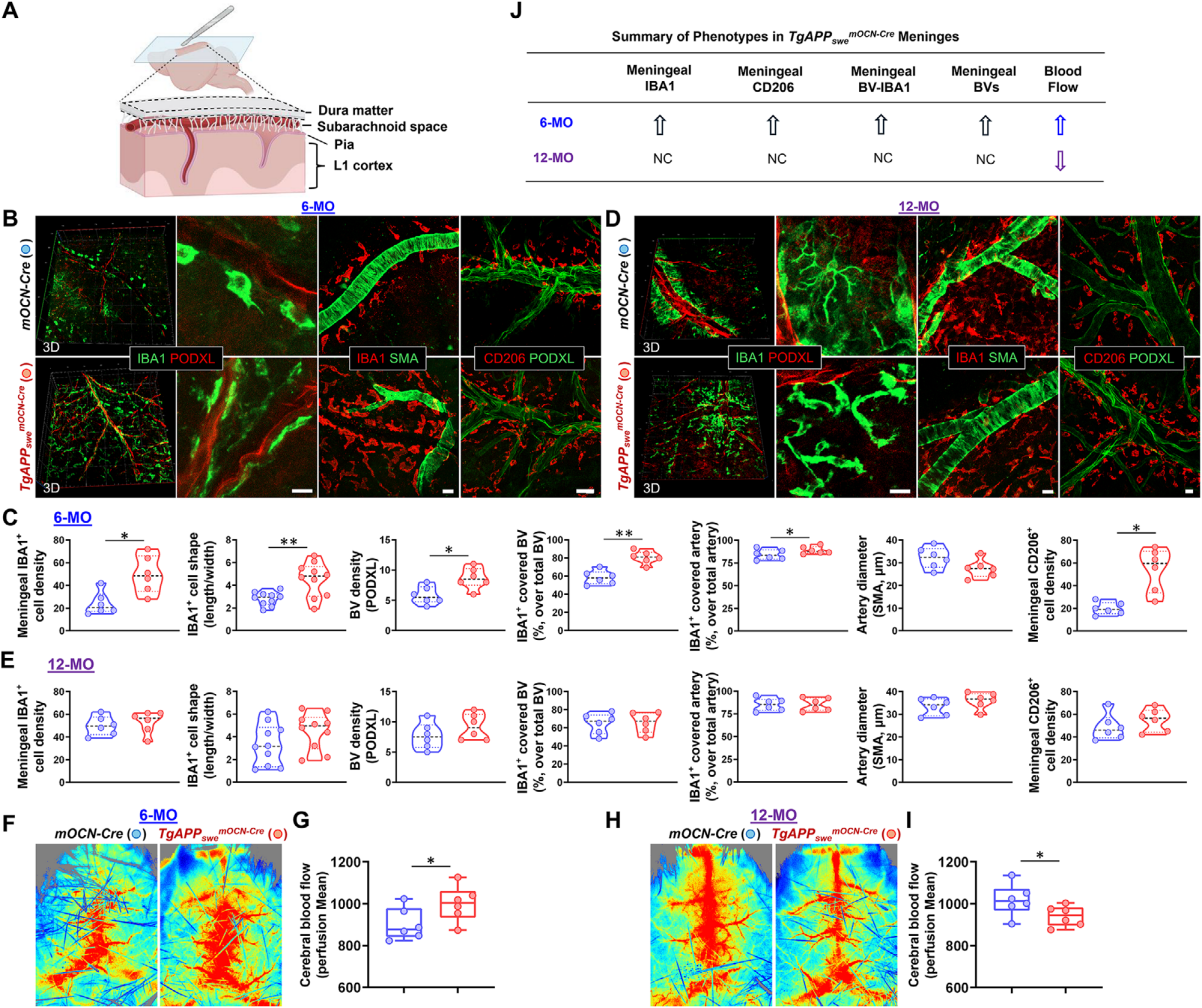
Age‐dependent changes in meningeal blood vessels and blood flow in Tg*APP_swe_^mOCN‐Cre^* mice. (A) Tissue cutting schematic. 6‐MO or 12‐MO *mOCN‐Cre* and *TgAPP_swe_^mOCN‐Cre^* mice were used for whole‐mount transverse tissue sectioning for cortical surface immunostaining. Tissue sections were ∼1 mm deep into the cortex and contained layers of the meninges such as the pia. (B) Representative images of cortical surface sections from 6‐MO *mOCN‐Cre; Ai9* and *TgAPP_swe_^mOCN‐Cre^; Ai9* mice coimmunostained with IBA1or CD206 and PODXL or SMA. Scale bar, 20 µm. (C) Quantification analyses of meninges IBA1^+^ cell density, IBA1^+^ cell shape, blood vessels (BV) density, percentage of IBA1^+^ cells covered BV, percentage of IBA1^+^ cells covered arteries, and artery diameter, and meningeal CD206^+^ cell density in B. (D) Representative images of cortical surface sections from 12‐MO *mOCN‐Cre; Ai9* and *TgAPP_swe_^mOCN‐Cre^; Ai9* mice coimmunostained with IBA1 or CD206 and PODXL or SMA. Scale bar, 20 µm. (E) Quantification analyses in D. (F) Representative image of unilateral cerebral blood flow analyses of 6‐MO *mOCN‐Cre* and *TgAPP_swe_^mOCN‐Cre^* mice via laser speckle contrast imaging. (G) Quantification of average recorded perfusion in F. (H) Representative image of unilateral cerebral blood flow analyses of 12‐MO *mOCN‐Cre* and *TgAPP_swe_^mOCN‐Cre^* mice via laser speckle contrast imaging. (I) Quantification of average recorded perfusion in H. (J) Summary of phenotypes in *TgAPP_swe_^mOCN‐Cre^* meninges. Data in (C) and (E) are shown as violin plots together with individual data points, and dot lines and dash line represent the quartiles and the median, respectively. Data in (G) and (I) are shown as box plots together with individual data points, and whiskers indicate the minimum to maximum. *n* = 6 male mice of each genotype. P values obtained by an unpaired two‐tailed *t*‐test. *P < 0.05. **P < 0.01.

To further investigate whether osteoblastic APP_swe_ expression regulates the migration of SBM‐derived myeloid cells to the dural and meningeal compartments, we examined IBA1^+^ macrophages and blood vessels (BVs) (PODXL^+^ or SMA^+^) in the meninges of control and *TgAPP_swe_^mOCN‐Cre^* mice at 6 and 12 months of age. To enable this analysis, we used a thin transverse sectioning approach to isolate the upper ∼1 mm of cortex along with the intact overlying pial and meninges for whole‐mount co‐immunostaining (Figure 3A). Strikingly, a significant increase in PODXL^+^ vessel‐associated IBA1^+^ cells [vessel‐associated macrophages (VAMs)] was detected in the meninges of 6‐MO *TgAPP_swe_^mOCN‐Cre^* mice (Figure 3B,C). These VAMs exhibited distinct morphological features, including reduced circularity and stronger association with both PODXL^+^ vessels and smooth muscle actin (SMA)^+^ arteries (Figure 3B,C), consistent with a migratory state. In parallel, the overall density of non‐arterial PODXL^+^ BVs appeared to be elevated in 6‐MO mutant meninges (Figure 3B,C). By 12 months, however, VAM density was no longer elevated in mutants compared to controls (Figure 3D,E). More IBA1^+^ cells in 12‐MO controls displayed a ramified morphology with star‐like processes, while most of those in 6‐MO mice had larger somas and fewer processes (Figure 3B,D). In 12‐MO mutant mice, IBA1^+^ cells retained the elongated morphology seen at 6‐MO but exhibited less association with small vessels (Figure 3D,E). Further co‐immunofluorescence staining using antibodies against CD206, a marker of M2‐like macrophages, together with IBA1 revealed an increased density of CD206^+^ cells in 6‐MO, but not 12‐MO, *TgAPP_swe_^mOCN‐Cre^* mice (Figure 3B–E). In addition, the proportion of IBA1^+^CD206^+^ double‐positive cells relative to total IBA1^+^ cells was significantly elevated in *TgAPP_swe_^mOCN‐Cre^* mice compared with controls (Figure S4A–F). Collectively, these findings indicate age‐dependent alterations in meningeal VAMs and BVs driven by osteoblastic APP_swe_ expression.

Given the dynamic changes of meningeal VAMs and BVs, we next examined whether meningeal blood flow was altered in 6‐ and 12‐MO *TgAPP_swe_^mOCN‐Cre^* mice. Intriguingly, Laser doppler imaging, which primarily detects superficial vascular perfusion in the meninges and upper cortex, revealed dynamic, age‐related changes (Figure 3F–I). At 6‐MO, blood perfusion or flow was significantly increased in the mutant mice compared to controls (Figure 3F,G). By 12 months, flow declined in mutants to levels below those of controls (Figure 3H,I). These blood flow changes closely paralleled the temporal pattern of meningeal VAM abundance (Figure 3J), suggesting an association between VAM dynamics and local vascular perfusion. Notably, CD206^+^ M2‐like macrophages are often associated with anti‐inflammatory and pro‐angiogenic functions, suggesting a potential role in modulating local vascular responses.

We further assessed glial cell responses in *TgAPP_swe_^mOCN‐Cre^* cortex, focusing on GFAP^+^ astrocytes and IBA1^+^ microglia, based on prior findings in *TgAPP_swe_^hOCN‐Cre^* mice [23]. Indeed, at 6‐MO, co‐immunostaining revealed an increase in IBA1^+^ microglia, predominantly in cortical layers I– IV, and elevated GFAP^+^ astrocytes localized to the pial membrane and Layer I (Figure S5A,B). By 12 months, astrocyte density remained elevated in these regions, whereas microglial density in Layers II– IV returned to control levels (Figure S5C,D). These glial cell responses were regionally specific to the cortex and absent in the hippocampus (Figure S5E–H), underscoring a spatially and temporally restricted effect of osteoblastic APP_swe_ expression on cortical glial activation.

Notably, similar to male mutants, female *TgAPP_swe_^mOCN‐Cre^* mice exhibited alterations in skull bone structure, meningeal features, and cerebral blood flow (CBF) relative to controls (Figure S6A–K). These findings suggest that the osteoblastic APP_swe_ driven changes in SBM–meninges–cortex axis is also largely preserved in female mice at this age.

Collectively, these findings reveal a transient increase in vascular‐associated glial cells, particularly IBA1^+^ cells, in the meninges and superficial cortex of *TgAPP_swe_^mOCN‐Cre^* mice. This glial activation corresponds closely with changes in meningeal blood flow and supports a model in which SBM‐derived myeloid cells access the meninges and cortical compartments via osteoblast‐modulated vascular pathways, which may be critical for modulating cerebral blood flow.

### 2.4 Similar Age‐Dependent Alterations in SBM, Meningeal VAMs, and Cerebral Blood Flow in *Tg2576* Mice

**FIGURE 4:**
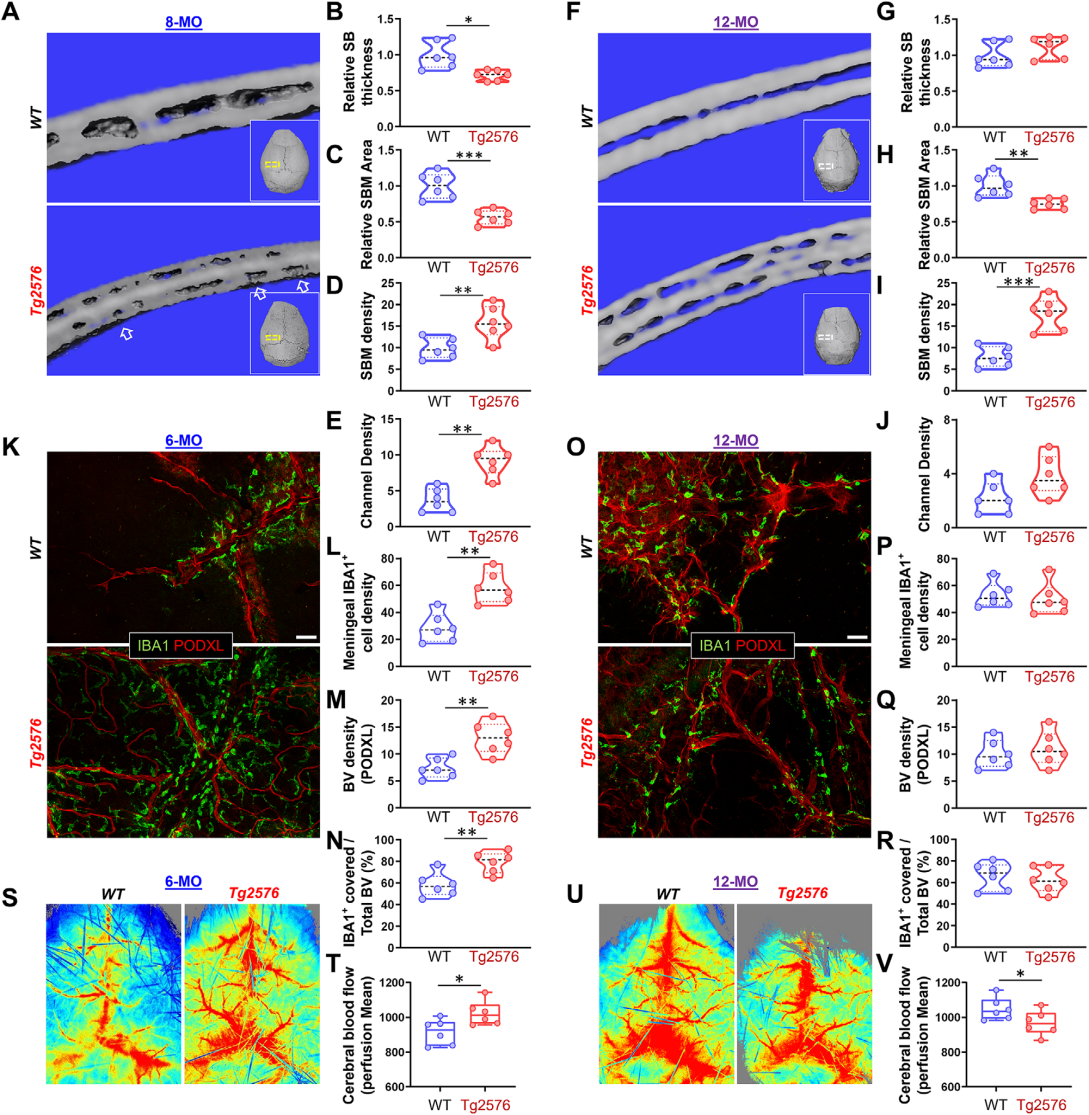
Altered skull bone remodeling in 6 to 8‐MO and 12‐MO *Tg2576* mice. (A) Representative µCT 3D images of skull bone from 8‐month male WT and *Tg2576* mice littermates. (B–E) Quantification analyses of SB thickness, SBM area, SBM density, and channel density in a by direct model of µCT analysis. (F) Representative µCT 3D images of skull bone from 12‐month male WT and *Tg2576* mice littermates. (G–J) Quantification analyses of SB thickness, SBM area, SBM density, and channel density in F by direct model of µCT analysis. (K) Representative images of cortical surface sections from 6‐MO WT and *Tg2576* mice coimmunostained with IBA1 and PODXL. Scale bar, 50 µm. (L–N) Quantification analyses of meninges IBA1^+^ cell density, BV density, and percentage of BV covered by IBA1^+^ cells in K. (O) Representative images of cortical surface sections from 12‐MO WT and *Tg2576* mice coimmunostained with IBA1 and PODXL. Scale bar, 50 µm. (P–R) Quantification analyses of meninges IBA1^+^ cell density, BV density, and percentage of BV covered by IBA1^+^ cells in O. (S) Representative image of unilateral cerebral blood flow analyses of 6‐MO WT and *Tg2576* mice via laser speckle contrast imaging. (T) Quantification of average recorded perfusion in S. (U) Representative image of unilateral cerebral blood flow analyses of 12‐MO WT and *Tg2576* mice via laser speckle contrast imaging. (V) Quantification of average recorded perfusion in U. Data in (B‐E), (G‐J), (L‐N), and (P‐R) are shown as violin plots together with individual data points, and dot lines and dash line represent the quartiles and the median, respectively. Data in (T) and (V) are shown as box plots together with individual data points, and whiskers indicate the minimum to maximum. *n* = 6 male mice of each genotype. P values obtained by unpaired two‐tailed *t*‐test. *P < 0.05. **P < 0.01. ***P < 0.001.

We further addressed whether similar SBM and meningeal changes could occur in other AD mouse models, such as *Tg2576* and *APP^NL‐G‐F^* mice. Tg2576 is a widely used transgenic line expressing hAPP_swe_ under the prion promoter, with broad tissue expression including bone [18], while *APP^NL‐G‐F^* is a knock‐in (KI) mouse model harboring humanized APP mutations [19]. Histological analysis of skull sections from both models revealed comparable alterations to those in *TgAPP_swe_^mOCN‐Cre^* mice, including thinner skull bone, reduced SBM volume, increased SBM densities in both 6‐ and 12‐MO (Figure S7). Importantly, further microCT imaging of *Tg2576* mice revealed similar but more severe SB phenotypes than those of *TgAPP_swe_^mOCN‐Cre^* mice, with more numerous small BMs distributed in two layers (Figure 4A–J), and the vascular‐like channels were increased at 6 to 8‐MO, but not at 12‐MO (Figure 4E,J). We also examined meningeal VAMs and blood flow in *Tg2576* mice. Again, similar age‐dependent alterations in both meningeal VAMs and blood flow were detected in *Tg2576* mice (Figure 4K–V). Together, these results are in line with the view for SBM‐derived myeloid cells to play a role in modulating cerebral blood flow and provide additional evidence that skull‐ and meninges‐related vascular remodeling may represent a common early structure feature across multiple AD models.

### 2.5 Age‐Dependent Cognitive Changes in *TgAPP_swe_^mOCN‐Cre^* Mice

To determine whether altered communications in SBM‐meninges‐cortex axis contribute to AD development, we asked if *TgAPP_swe_^mOCN‐Cre^* mice show an age‐dependent cognition changes, similar to those in *Tg2576* and *TgAPP_swe_^hOCN‐Cre(tg)^* mice. To this end, we subjected *TgAPP_swe_^mOCN‐Cr^* ^e^ and control (*mOCN‐Cre*) mice to a battery of behavioral tests at 6‐ and 12‐MO. These included the open field test (OFT) to assess anxiety‐like behavior and locomotor activity, the Morris water maze (MWM) for spatial learning and memory, the novel object recognition (NOR) test for recognition memory, and the Y‐maze for working memory.

In the OFT, *TgAPP_swe_^mOCN‐Cre^* mice at both ages spent significantly less time in the center of the arena compared to age‐matched controls, despite similar total distances traveled (Figure S8). These findings indicate reduced exploratory behavior in the mutants, suggestive of increased anxiety‐ and/or depression‐like tendencies, without impairments in general locomotion.

**FIGURE 5:**
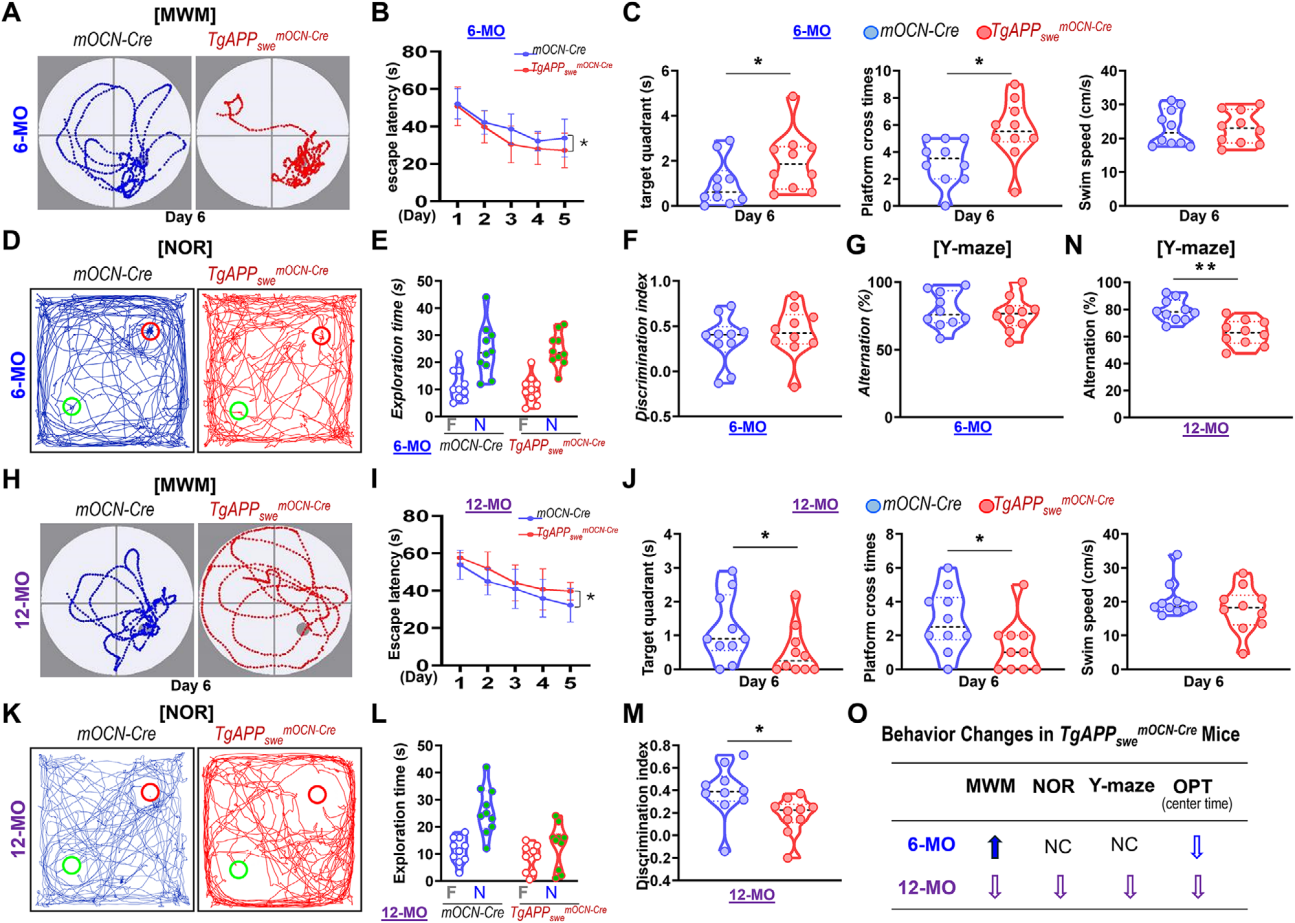
Age‐dependent cognition declines by *TgAPP_swe_^mOCN‐Cre^* mice. (A–C) 6‐MO *mOCN‐Cre* and *TgAPP_swe_^mOCN‐Cre^* male mice were subject to Morris water maze (MWM), the latencies to reach the hidden platform during the training period were shown in B, and the representative tracing images and quantification of time spent in the target quadrant, platform crossing time, and swim speed were shown in A and C. (D–F) Novel Object Recognition (NOR). The time spent with familiar and novel object was shown in E. The discrimination index which is calculated by subtracting the exploration time of the familiar object from the exploration time of the novel object and then dividing by the total exploration time for both objects, is shown in F. (G) Y‐maze test. Spontaneous alternation was shown. (H–N) 12‐MO *mOCN‐Cre* and *TgAPP_swe_^mOCN‐Cre^* male mice were subject to MWM (h‐j), NOR (K‐M), and Y‐maze (N) tests. (O) Summary of behavior changes in 6‐MO and 12‐MO *TgAPP_swe_^mOCN‐Cre^* mice. Data in (B) and (I) are presented as mean± SD (*n* = 10). Data in (C), (E‐G), (J), and (L‐N) are shown as violin plots together with individual data points, and dot lines and dash line represent the quartiles and the median, respectively. (*n* = 10 animals per genotype). *P* values obtained by one‐way ANOVA followed by Tukey post hoc test in (B) and (I), and unpaired two‐tailed *t*‐test in the others. *P < 0.05.

Critically, age‐dependent differences emerged in cognitive task performance across MWM, NOR, and Y‐maze tests. At 6‐MO, *TgAPP_swe_^mOCN‐Cre^* mice exhibited slightly enhanced spatial learning and long‐term memory in the MWM (Figure 5A–C), with no significant differences observed in the NOR or Y‐maze (Figure 5D–G) compared to controls. However, by 12‐MO, *TgAPP_swe_^mOCN‐Cre^* mice displayed significant impairments across all three behavioral paradigms (Figure 5H–N).

These results highlight a clear age‐dependent trajectory of cognitive function in *TgAPP_swe_^mOCN‐Cre^* mice (Figure 5O), mirroring patterns observed in *TgAPP_swe_^hOCN‐Cre(tg)^* and *Tg2576* mice [23, 31, 32]. Together with our earlier findings, this raises an intriguing question about whether the observed age‐related changes in SBM, meningeal VAMs, cortical microglia, and cerebral blood flow are mechanistically linked to cognition changes.

### 2.6 BMM Transplantation Enhances Recruitment of IBA1^+^ Cells to SBM, Meninges, and Cortex, and Improves Cerebral Blood Flow and Cognitive Function in 12‐MO *TgAPP_swe_^mOCN‐Cre^* Mice

**FIGURE 6:**
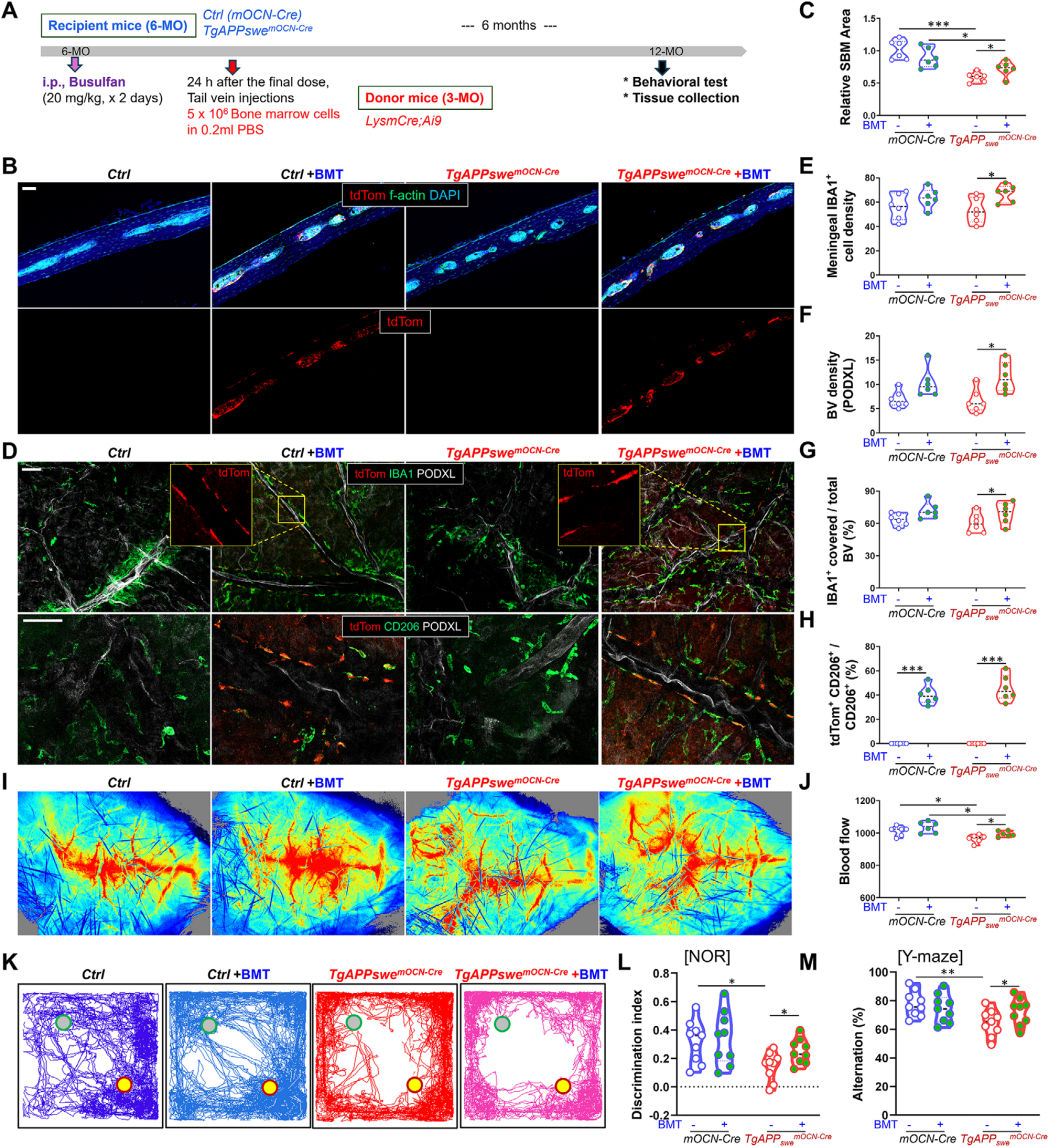
Bone marrow macrophage transplantation promotes myeloid cells from blood to SBM, meningeal, and cortex, enhances cerebral blood flow, and improves cognitive function in 12‐MO *TgAPP_swe_^mOCN‐Cre^* mice. (A) Illustration of Bone marrow macrophage transplantation. (B) Representative images of skull bone sections from 12‐MO *mOCN‐Cre; Ai9* and *TgAPP_swe_^mOCN‐Cre^; Ai9* mice with or without BMT coimmunostained with f‐actin (green) and DAPI (blue). Cre‐positive BM cells express the tdTomato (red). Scale bar, 100 µm. (C) Quantification analyses of SBM area in B. (D) Representative images of cortical surface sections coimmunostained with PODXL and IBA1 or CD206. Scale bar, 50 µm. (E–H) Quantification analyses of meninges IBA1^+^ cell density, BV density, percentage of BV length covered by IBA1^+^ cells, and percentage of tdTom^+^ CD206^+^ of total CD206^+^ in D. (I) Representative image of unilateral cerebral blood flow analyses via laser speckle contrast imaging. (J) Quantification of average recorded perfusion in I. (K,L) Novel Object Recognition (NOR). The discrimination index was shown in L. (M) Y‐maze test. Spontaneous alternation was shown. Data in (C), (E‐H), (J), and (L‐M) are shown as violin plots together with individual data points, and dot lines and dash line represent the quartiles and the median, respectively. (*n* = 6 to 9 male animals per genotype). *P* values obtained by two‐way ANOVA followed by Bonferroni's post hoc test. *P < 0.05. **P < 0.01. ***P < 0.001.

To address the question above, we investigated whether bone marrow macrophage transplantation (BMMT) could enhance macrophage presence in the SBM and meninges, thereby influencing cerebral blood flow and cognitive function. For this purpose, bone marrow cells were isolated from the femoral bone marrow of 3‐MO *Lyz‐M‐Cre;Ai9* mice, in which macrophages express tdTomato fluorescence (Figure 6A). These tdTomato^+^ BMMs were transplanted into 6‐MO *TgAPP_swe_^mOCN‐Cre^* and control (mOCN‐Cre) recipient mice (Figure 6A). Six months following BMMT, the mice underwent cognitive behavioral testing and were then sacrificed for immunohistological analysis (Figure 6A). As shown in Figure 6B–H, tdTomato^+^ macrophages were detectable in the SBM, meninges, and cortical surface of 12‐MO *TgAPP_swe_^mOCN‐Cre^* and control mice following BMMT. Notably, abundant tdTomato^+^CD206^+^ double‐positive cells were observed in the meninges, indicating the presence of transplanted macrophages with an M2‐like phenotype in this compartment (Figure 6D,H). Interestingly, BMMT enhanced cerebral blood flow and improved performance in the novel object recognition (NOR) test and Y‐maze test in *TgAPP_swe_^mOCN‐Cre^* mice (Figure 6H–M). These findings support the view that BM–derived myeloid cells/macrophages, through the SBM–meninges–cortex pathway, play a role in enhancing cerebral perfusion and cognitive function.

### 2.7 RNAseq Analysis Showing Changes in Multi‐Pathways, Including Down‐Regulation of CELL Proliferation, in APP_swe_^+^ Calvarial Osteoblasts

We next investigated how APP_swe_^+^ osteoblasts affect SB and SBM remodeling. To this end, we isolated tdTomato^+^ calvarial osteoblasts from the skulls of *TgAPP_swe_^mOCN‐Cre^;Ai9* mice and control (*mOCN‐Cre;Ai9)* mice at 6‐MO using fluorescence‐activated cell sorting (FACS) and subjected them to bulk RNA sequencing (Figure S9A). Transcriptomic profiling revealed 425 upregulated and 478 downregulated genes in APP_swe_‐expressing osteoblasts compared to controls (Figure S9B). Gene ontology (GO) pathway analysis showed that the upregulated genes were primarily enriched in pathways related to positive regulation of cell migration, cytokine production, Wnt/β‐catenin signaling, angiogenesis, cell‐cell adhesion, and negative regulation of cell proliferation (Figure S9C), while downregulated genes were associated with cell‐cell signaling, cell proliferation, and osteoclast differentiation (Figure S9C). Heatmap analysis further highlighted differentially expressed secreted genes associated with wnt‐protein binding, cell adhesion, and cell proliferation (Figure S9D).

### 2.8 Age‐Dependent Reductions in SB and SBM Cell Proliferations in *TgAPP_swe_^mOCN‐Cre^* Mice

**FIGURE 7:**
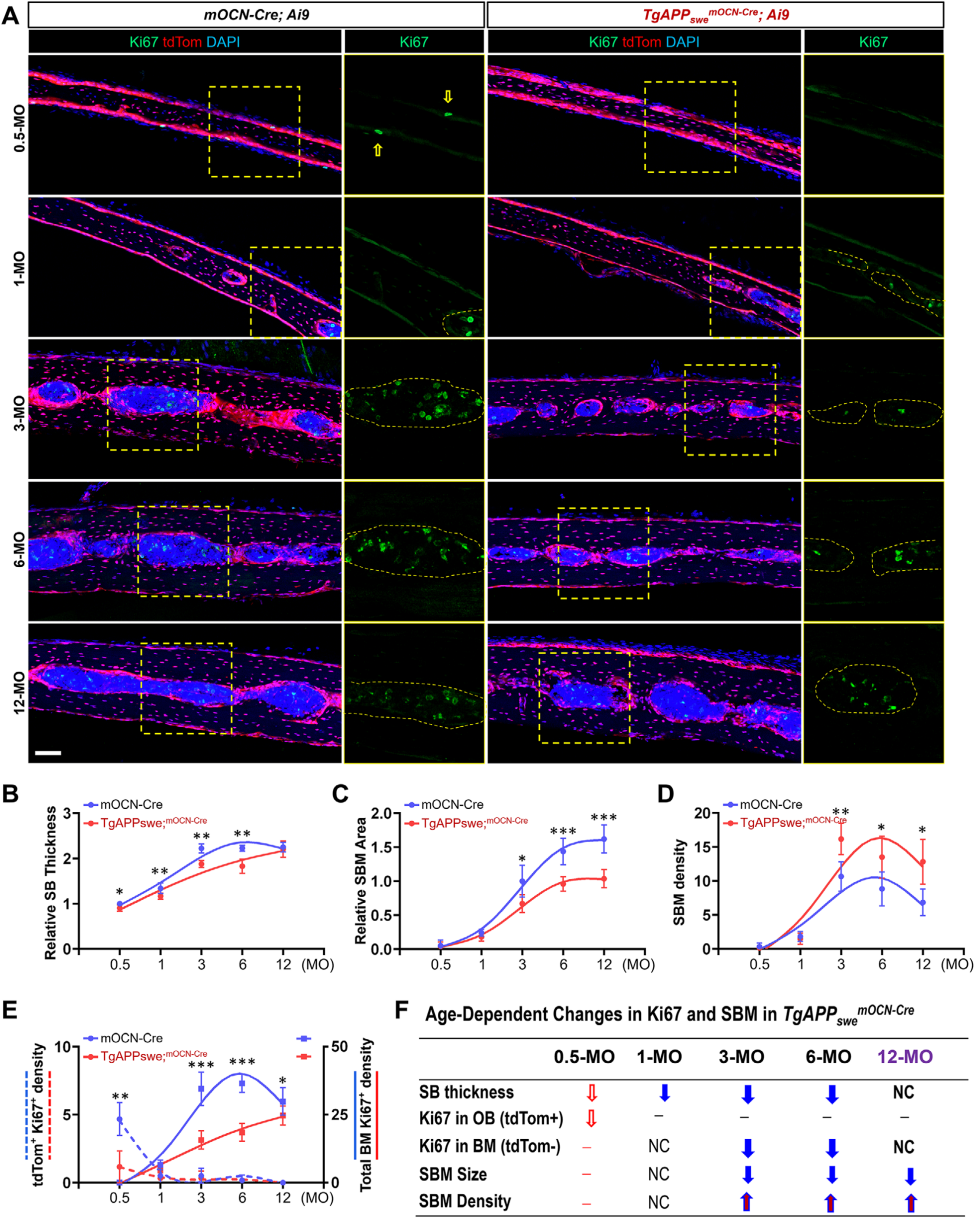
Changed skull bone remodeling in *TgAPP_swe_^mOCN‐Cre^* mice during development. (A) Representative images of skull bone sections from 0.5MO, 1‐MO, 3‐MO, 6‐MO, and 12‐MO *mOCN‐Cre; Ai9* and *TgAPP_swe_^mOCN‐Cre^; Ai9* mice coimmunostained with Ki67 (green) and DAPI (blue). Cre‐positive cells express the tdTomato (red). Scale bar, 100 µm. (B–E) Quantification analyses of SB thickness, SBM area, SBM density, and Ki67^+^ cell density in A. (F) Summary of age‐dependent changes in Ki67 and SBM in *TgAPP_swe_^mOCN‐Cre^* mice. Data in (B‐E) are presented as mean± SD (*n* = 6). Statistical analysis was performed using one‐way ANOVA followed by Tukey's post hoc test. *P < 0.05. **P < 0.01. ***P < 0.001.

To determine the functional relevance of these transcriptomic changes, we examined skull bone cell proliferation across developmental stages. At P15 (0.5 MO), prior to SBM formation, the skull bones appeared grossly normal in mutant mice, though a slight reduction in SB thickness was observed (Figure 7A,B). This was accompanied by a decrease in mOCN‐Cre^+^ proliferating cells (tdTom^+^Ki67^+^), primarily localized in the dura mater and periosteum (Figure 7A–E), suggesting that early thinning of the skull may result from reduced osteoblast proliferation.

By ∼1‐MO, BM began to form in the skull and progressively expanded with age (Figure 7A,C,D). SB thickness and SBM density peaked around 3‐MO (Figure 7B,D), while SBM volume continued to increase thereafter (Figure 7C). In *TgAPP_swe_^mOCN‐Cre^* mice, no major changes in SBM were detected at 1‐MO; however, starting at 3‐MO, clear alterations emerged, including reduced SBM volume and increased SBM density (Figure 7A,C,D).

Interestingly, tdTom^+^Ki67^+^ proliferative osteoblasts were rarely observed beyond 1‐MO (Figure 7A,E, dotted lines), indicating that skull bone cell proliferation is largely restricted to early postnatal stages. In contrast, Ki67^+^ cells within the BM, representing tdTom^−^ marrow‐resident cells, persisted into later ages, peaked in 3‐ to 6‐MO, declined in 12‐MO, which were significantly reduced in *TgAPP_swe_^mOCN‐Cre^* mice (Figure 7A,E, continuous lines).

Collectively (Figure 7F), these findings reveal age‐dependent changes in the SBM in response to osteoblast‐specific APP_swe_ expression and suggest both cell‐autonomous effects (e.g., reduced osteoblast proliferation contributing to SB thinning) and cell non‐autonomous effects (e.g., altered proliferation and composition of SBM cells).

### 2.9 Necessity of osteoblastic ATP6AP2 for SBM formation, enhanced myeloid/microglial cell trafficking to meningeal/cortex, and improved cerebral blood flow and cognition, in 6‐MO *TgAPP_swe_^mOCN^* ^‐Cre^ mice

To further investigate the contribution of SBM remodeling to AD pathogenesis and to elucidate how osteoblastic APP_swe_ influences SBM remodeling, we focused on ATP6AP2 for several reasons. First, ATP6AP2 is a key regulator of Wnt/β‐catenin signaling in various cell types, including osteoblasts, a pathway essential for bone formation and bone marrow (BM) remodeling (Figure S10A,B) [33, 34, 35, 36]. Second, it functions as a co‐factor for v‐ATPase assembly, thereby regulating lysosomal activity [37, 38], a process often disrupted in neurodegenerative diseases [39]. Third, dysfunction of ATP6AP2 has been implicated in patients with neurodegeneration and dementia [40, 41, 42, 43]. Finally, comparative analysis of our RNA‐seq data revealed differential regulation of ATP6AP2 transcripts in APP_swe_^+^ osteoblasts: expression was reduced in femoral long bones but showed little change in skull bones [23]. In contrast, Western blot analysis demonstrated a slight increase in ATP6AP2 protein levels in APP_swe_^+^ skull bone osteoblasts (Figure S10C,D).

As RNA‐seq analysis revealed no significant change in ATP6AP2 transcript levels (Figure S9), suggesting that APP_swe_ does not regulate ATP6AP2 at the transcriptional level. Given that both APP and ATP6AP2 are membrane‐associated proteins localized to vesicular compartments, we next examined their subcellular distribution. Co‐immunostaining in MC3T3 osteoblastic cells expressing APP_WT_‐YFP or APP_swe_‐YFP showed weak co‐localization between APP_WT_ and ATP6AP2, whereas APP_swe_ exhibited markedly increased co‐localization with ATP6AP2, primarily within LAMP1^+^ late endosome/lysosome compartments (Figure S10E–I). Consistently, ATP6AP2 displayed limited association with LAMP1^+^ vesicles in APP_WT_‐expressing cells but was enriched in these compartments in APP_swe_‐expressing cells (Figure S10E–I). Notably, LAMP1^+^ vesicles appeared enlarged in APP_swe_‐expressing cells, and a subset of APP_swe_ co‐localized with both ATP6AP2 and LAMP1 in aggregated structures (Figure S10I). These findings suggest that APP_swe_ alters the subcellular distribution of ATP6AP2 and is associated with changes in lysosomal compartments.

**FIGURE 8:**
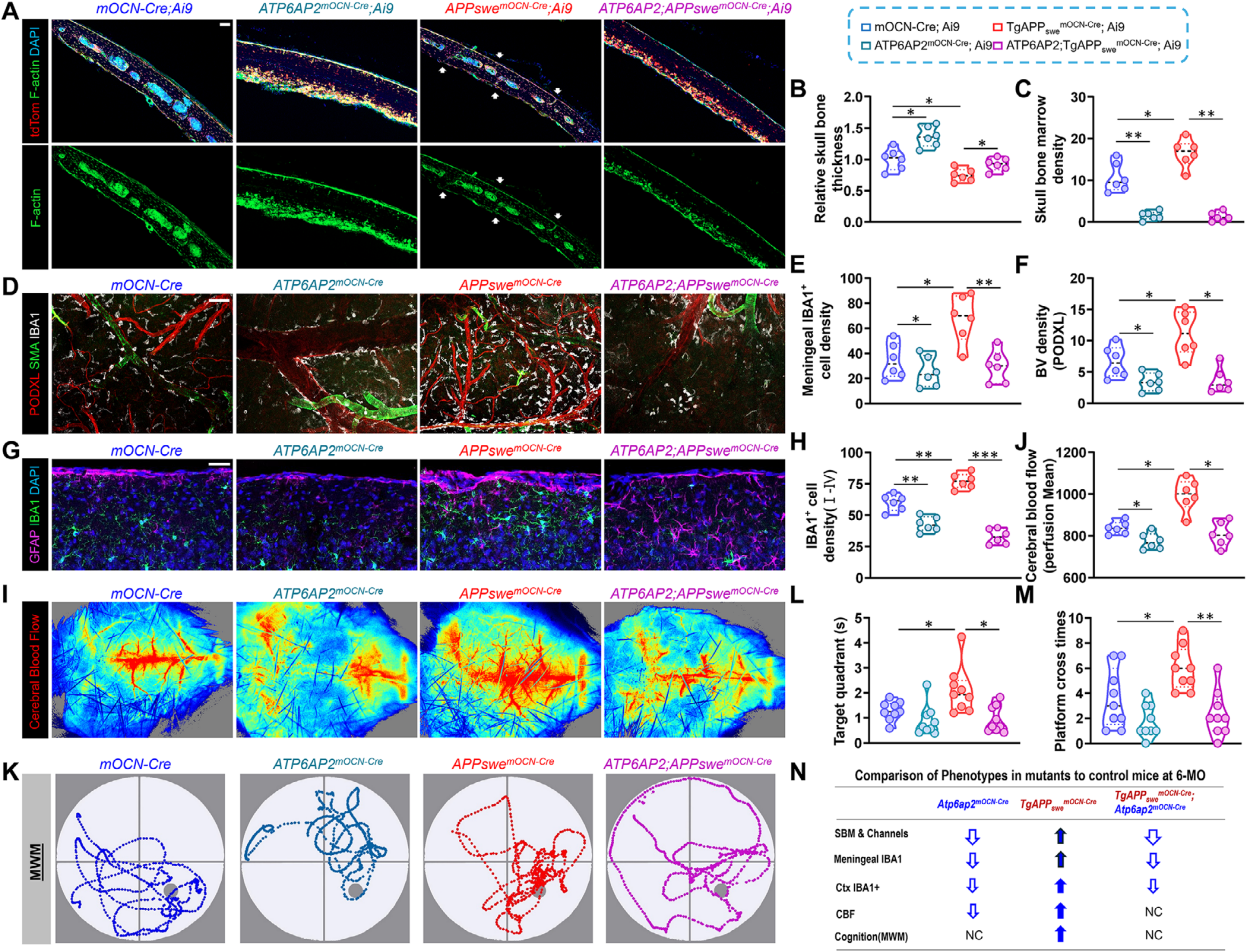
Osteoblastic ATP6AP2 is essential for SBM and channel formation, necessary for the increased myeloid/microglial cells in meningeal/cortex, blood flow, and cognitive improvement in 6‐MO *TgAPP_swe_^mOCN‐Cre^* mice. (A–C) Representative images of skull bone sections from 6‐MO *mOCN‐Cre; Ai9, ATP6AP2^mOCN‐Cre^; Ai9, TgAPP_swe_^mOCN‐Cre^; Ai9*, and ATP6AP2;*TgAPP_swe_^mOCN‐Cre^; Ai9* mice coimmunostained with f‐actin (green) and DAPI (blue). Cre‐positive cells express the tdTomato (red). Scale bar, 100 µm. Quantification analyses of SB thickness and SBM density are presented in B and C. (D–F) Representative images of cortical surface sections coimmunostained with IBA1, PODXL, and SMA. Scale bar, 50 µm. Quantification analyses of meninges IBA1^+^ cell density, BV density are prescented in E and F. (G,H) Representative images of co‐immunostaining with IBA1 (green), GFAP (magenta), and DAPI (blue) of cortex sections. Scale bar, 50 µm. Quantification analyses of IBA1^+^ cell density (layer I‐IV) is presented in H. (I,J) Representative image of unilateral cerebral blood flow analyses via laser speckle contrast imaging. Quantification of average recorded perfusion is presented in J. (K–M) Mice were subject to Morris water maze (MWM), the representative tracing images, quantification of time spent in target quadrant, and platform crossing time were shown in K, L, and M. (N) Summary of phenotypes in mutants to control mice at 6‐MO. Data in (B‐C), (E‐F), (H), (J), and (L‐M) are shown as violin plots together with individual data points, and dot lines and dash line represent the quartiles and the median, respectively. *n* = 6 to 9 male mice of each genotype. Statistical analysis was performed using one‐way ANOVA followed by Tukey's post hoc test. *P < 0.05. **P < 0.01. ***P < 0.001.

To investigate osteoblastic ATP6AP2's function in SK and SBM development and remodeling, we generated *Atp6ap2^mOCN‐Cre^;Ai9* mice by crossing *Atp6ap2^f/w^* with *mOCN‐Cre;Ai9* and examined their skull bone phenotypes. Strikingly, 6‐MO *Atp6ap2^mOCN‐Cre^;Ai9* mice exhibited severe skull bone abnormalities, including marked reduction or absence of SBM and dural vascular channels, along with an accumulation of abnormal mOCN‐Cre^+^ (tdTom^+^) cells in the dura matter (Figure 8A–C). These findings highlight a previously unappreciated role for osteoblastic ATP6AP2 in the development and maintenance of SBM, providing a powerful genetic model to further dissect the function of the SBM in brain myeloid cell homeostasis, cerebral blood flow, and cognitive function.

Building on these results, we crossed *Atp6ap2^mOCN‐Cre^; Ai9* mice with *TgAPP_swe_^mOCN‐Cre^* mice to generate double mutants. Remarkably, the double mutant animals phenocopied the *Atp6ap2^mOCN‐Cre^* mice, exhibiting near‐complete loss of SBM and dural channel formation, as well as a pronounced reduction in IBA1^+^ myeloid/microglial cells in both the meninges and cortex (Figure 8A–H). The GFAP^+^ astrocytes in the double mutant cortex remain elevated (Figure 8G). Importantly, both single (*Atp6ap2^mOCN‐Cre^*) and double (*Atp6ap2^mOCN‐Cre^; TgAPP_swe_^mOCN‐Cre^*) mutant mice showed significant impairments in cortical blood flow, as measured by laser Doppler imaging (Figure 8I,J), and deficits in cognitive performance, assessed by the MWM, NOR and Y‐maze tests (Figure 8K–M; Figure S11).

Collectively (Figure 8N), these findings demonstrate the critical role of ATP6AP2 in SB and SBM development, emphasize the SBM as a cellular reservoir, and SBM‐to‐dura channels as essential conduits for the migration of myeloid cells into the cortex. Furthermore, these results provide additional evidence for a potential contribution of these myeloid/microglial populations to the regulation of cerebral blood flow and cognitive function, establishing a novel bone‐to‐brain communication axis relevant to aging and neurodegeneration.

## 3 Discussion

**FIGURE 9:**
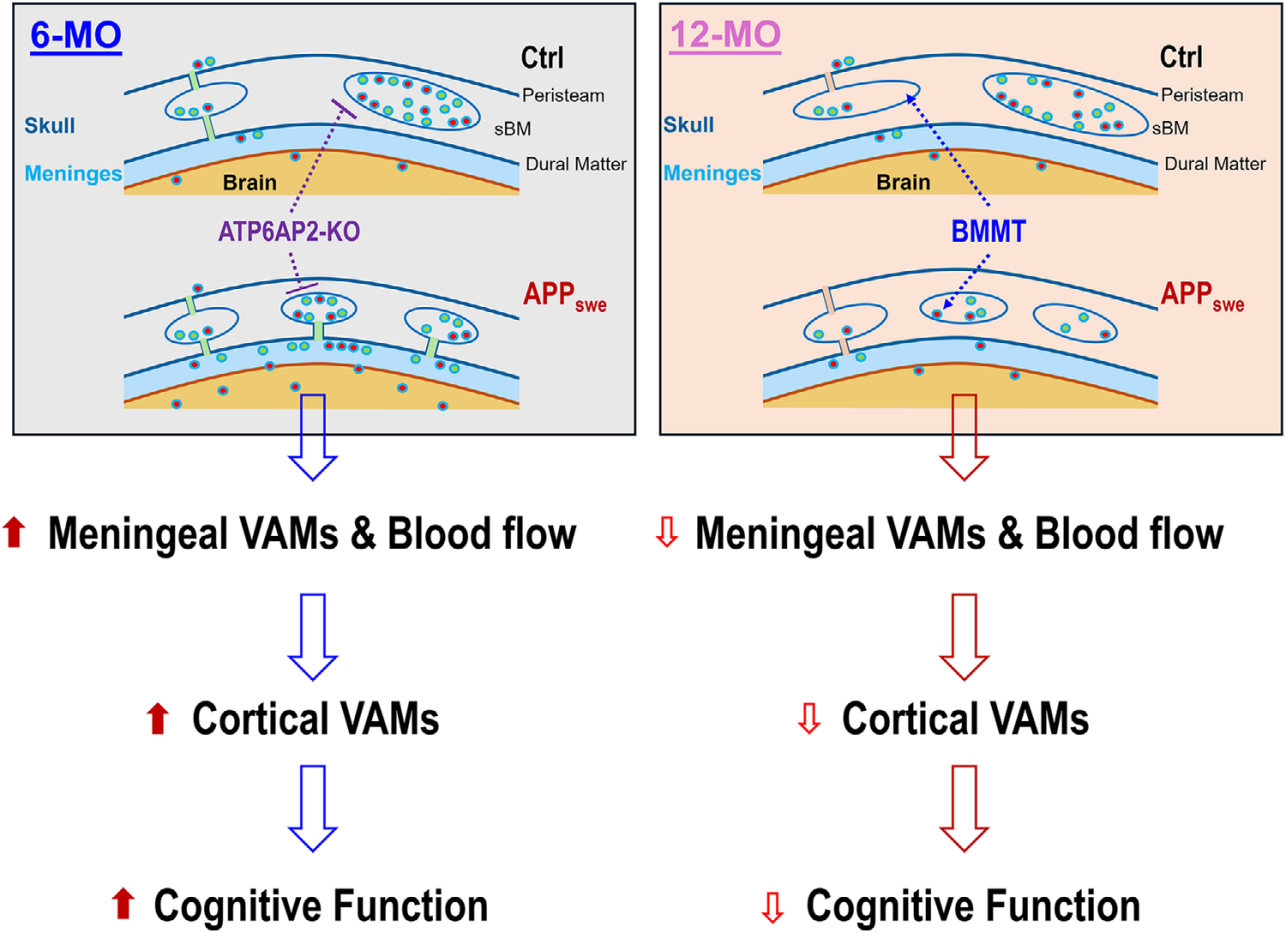
Working model illustrating how osteoblastic APPswe regulates SBM–meningeal communication and brain function across aging. Osteoblastic APP_swe_ expression exerts an early, age‐dependent, and cell non‐autonomous influence on skull bone marrow (SBM) remodeling and the associated vascular channels that connect SBM to the dural meninges. In young adult mice (6‐MO), APP_swe_ expression increases SBM–meningeal channels, which correlates with enhanced migration of meningeal vessel‐associated macrophages (VAMs) into the meninges and cortex, elevated cerebral blood flow, and improved cognitive performance. By 12‐MO of age, these channels decline. Mechanistically, these effects require osteoblastic ATP6AP2, a key regulator of Wnt/β‐catenin signaling. Osteoblastic deletion of ATP6AP2 disrupts SBM–brain communication, reduces cerebral blood flow, and results in cognitive decline. Conversely, bone marrow macrophage transplantation (BMMT) in aged *TgAPP_swe_^mOCN‐Cre^* mice restores SBM–meningeal communication, rescuing cerebral perfusion and cognitive function.

The discovery of SBM–meningeal channels [44, 45, 46], has opened new directions in neuro‐immune research but raises key questions: What brain functions does SBM–meninges communication regulate? How do AD risk factors influence it? Does its disruption drive neurodegeneration? How is SBM remodeling controlled, and can peripheral cells like osteoblasts modulate this axis? To address these questions, we developed a TgAPP_swe_^mOCN‐Cre^ mouse model expressing APP_swe_ specifically in osteoblasts. Our findings reveal an altered SBM–brain axis in AD. First, early SBM changes, such as reduced cellularity, increased density, and expanded vascular channels, occur in both osteoblast‐specific and global APP_swe_ based AD models (e.g., Tg2576, APP^NL‐G‐F^). These phenotypes were also consistent in female 6‐MO mutants, although behavioral analyses were limited by sample size, suggesting the SBM–meninges–cortex axis may operate in both sexes while potential sex‐dependent cranial differences could contribute to variability [47]. Second, APP_swe_‐driven expansion of channels enhances migration of SBM‐derived myeloid cells into the meninges and cortex, which is associated with improving cerebral blood flow (CBF) and slowing cognitive decline. Third, boosting this axis via bone marrow cell transplantation improves CBF and cognition in aged mice. Finally, disrupting the axis through osteoblastic deletion of ATP6AP2 impairs both outcomes (Figure 9). Collectively, these results identify osteoblast‐driven SBM remodeling as a key regulator of meningeal and cortical immunity, vascular function, and cognition, highlighting a previously unrecognized systemic contribution to AD pathogenesis.

Notably, APP_swe_‐induced alterations in the SBM–brain axis, such as expanded vascular channels and increased distribution of SBM‐derived myeloid cells into the meninges and cortex, are age‐dependent. These changes are prominent at 6‐MO but diminish by 12 MO, coinciding with transient improvements in CBF and cognitive function. This temporary “enhancement” of the SBM–meninges–cortex axis at 6 MO may reflect an early compensatory response that delays the onset of brain and dementia‐related phenotypes. Over time, however, this compensation may accelerate depletion of SBM cells, particularly in the context of age‐associated senescence, ultimately contributing to the cognitive decline observed at 12 MO.

These observations are intriguing and raise the question of how osteoblastic APP_swe_ drives such age‐dependent changes in SBM. Specifically, how does osteoblastic APP_swe_ increase SBM channels in 6‐MO? Several potential mechanisms may explain its effects. First, direct interactions with SBM‐resident cells: APP_swe_ expression in osteoblasts may modulate their interaction with nearby SBM‐resident myeloid cells, influencing SBM remodeling and vascular channel formation. This, in turn, could facilitate myeloid cell migration to the meninges and cortex. Supporting this, we observed increased IBA1+/LY6C+ myeloid cell association with TdTomato+ osteoblasts at 6 months of age (6‐MO), but a reduction at 12‐MO, in APP_swe_ mutant skulls (Figure 2; Figure S2). These patterns closely match the distribution of vessel‐associated macrophages in the meninges (Figure 3). Second, changes in osteoblast‐derived secreted factors: APP_swe_ may alter the osteoblast secretome in an age‐dependent manner, affecting bone marrow stem/progenitor cell proliferation, differentiation, and migration. RNA‐seq analysis revealed APP_swe_‐induced gene expression changes involving Wnt/β‐catenin signaling, angiogenesis, and pro‐inflammatory pathways (Figure S9). These secreted factors likely contribute to reduced BM cell proliferation and altered lineage fate (Figure 7; Figure S9). Moreover, alterations in cytokine production (e.g., Il6, Il1rl2, Tnfsf15, Nlrp1b) and angiogenic regulators (e.g., Vegfa, Hgf, Mmp9, Ccr2) may further modulate SBM remodeling and channel formation, shaping the local microenvironment and influencing myeloid cell behavior and migration. Third, lysosomal regulation and ATP6AP2: APP_swe_ increases ATP6AP2 protein levels in skull bone osteoblasts without affecting transcript levels, suggesting post‐transcriptional regulation. APP_swe_ co‐localizes with ATP6AP2 in LAMP1^+^ late endosome/lysosome compartments, and LAMP1‐positive vesicles are enlarged in APP_swe_‐expressing cells, whereas APP_WT_ shows only weak co‐localization. These findings suggest that APP_swe_ may impair lysosomal trafficking or protein degradation, thereby altering the secretion of lysosome‐ or vesicle‐derived factors. Such changes could contribute to remodeling of the SBM microenvironment and trans‐osseous channels, promoting myeloid cell migration.

What accounts for the decline in osteoblastic APP_swe_–induced SBM changes in 12‐month‐old (MO) mice? Based on our findings and prior literature, we speculate that APP_swe_‐induced osteoblast senescence drives several interconnected processes in aged mutant mice, including reduced SBM progenitor populations, a diminished myeloid cell reservoir, and remodeling of the local microenvironment through increased secretion of senescence‐associated secretory proteins (SASPs). This interpretation is supported by our observation of a marked decline in cell proliferation within the SBM of 12‐MO mice (Figure 7) and is consistent with our previous report [23], in which clearance of senescent cells using dasatinib plus quercetin (D+Q) attenuated cortical brain phenotypes in mutant mice. However, whether senescent cell clearance similarly improves meningeal and CBF phenotypes—and whether such interventions confer greater benefit in older (12‐MO) versus younger (6‐MO) mutant mice—remains to be determined.

An additional question is whether the SBM phenotypes induced by osteoblastic APP_swe_ are mediated by Aβ production or by APP_swe_‐dependent, Aβ‐independent mechanisms. Although *TgAPP_swe_^mOCN‐Cre^* mice likely produce human Aβ in osteoblasts, prior ELISA measurements indicate that Aβ40/42 levels in bone marrow–derived cells are substantially lower than those in the brains of Tg2576 mice [23]. Moreover, similar skeletal deficits are observed in APP knockout mice [22], suggesting that the bone phenotypes may arise primarily from a dominant‐negative effect of APP_swe_ on endogenous APP rather than from Aβ itself. We therefore speculate that APP_swe_‐dependent, Aβ‐independent mechanisms contribute substantially to the observed age‐dependent SBM remodeling phenotypes.

BMMT has been shown to improve cognitive outcomes in several AD models [48, 49, 50]; and our studies suggest that via enhancing SBM–meninges–cortex communication, BMMT could improve cerebral perfusion and cognitive function in aged *TgAPP_swe_^mOCN‐Cre^* mice (Figure 6), revealing a potential mechanism underlying BMMT's therapeutic effect. However, notice that our BMMT experiments represent a preventive, proof‐of‐concept intervention rather than a therapeutic approach for established AD pathology. While numerous studies have shown that early bone marrow transplantation can modulate neuroinflammation, enhance amyloid clearance, and improve cognitive performance in AD mouse models [50], further work is required to test whether interventions targeting the SBM–meninges–cortex axis can reverse or halt disease progression once pathology is established. Together, these results align with growing evidence that SBM‐derived myeloid cells, as well as vascular‐associated macrophages and microglia, may play protective roles in various neuropathologies, including stroke, MS, and AD [1, 27, 28, 29, 30], and support the idea that systemic and skeletal factors can modulate neurovascular and neuroimmune functions relevant to AD pathogenesis.

How is SBM‐derived myeloid cell distribution linked with CBF? We propose several potential mechanisms. SBM‐derived cells that migrate to the meninges and perivascular spaces may differentiate into macrophages that regulate vascular tone through the release of vasoactive mediators, including nitric oxide, prostaglandins, and reactive oxygen species [29, 45, 51]. In addition, these immune cells may interact with endothelial cells, pericytes, and astrocytes within the neurovascular unit, modulating blood–brain barrier integrity and vascular reactivity [29, 52, 53]. Altered trafficking of myeloid cells may also shift the balance between pro‐ and anti‐inflammatory signals, leading to inflammation‐mediated vascular dysfunction [1, 45]. Finally, meningeal and perivascular macrophages can influence fluid drainage and waste clearance, indirectly affecting intracranial pressure and blood flow dynamics [52, 54]. These mechanisms are likely to act in concert to mediate CBF regulation by SBM‐derived myeloid cells, though direct testing will be required in future studies.

How does osteoblastic APP_swe_ regulate ATP6AP2? Our studies suggest that APP_swe_ may impair LAMP1^+^ lysosomal function, thereby reducing ATP6AP2 degradation and increasing its protein levels (Figure S10). Another important question is how does osteoblastic ATP6AP2 regulate SBM formation during development? ATP6AP2, also known as the (pro)renin receptor (PRR), is a key modulator of Wnt/β‐catenin signaling and vacuolar H^+^‐ATPase (v‐ATPase) function [33, 34, 35, 36]. Importantly, mutations in the human ATP6AP2 gene are linked to neurodevelopmental and neurodegenerative disorders with cognitive impairment [40, 41, 42, 43] However, the mechanisms underlying ATP6AP2 regulation of cognition remain elusive. We have previously demonstrated that osteoblastic ATP6AP2 deletion impairs Wnt/β‐catenin signaling in femoral bone and cultured osteoblasts, resulting in reduced bone formation and decreased bone mass [35]. Given the established role of Wnt/β‐catenin signaling in SBM development and remodeling [55, 56], and the dependence of this signaling pathway on ATP6AP2 [33, 34, 35], we propose that osteoblastic ATP6AP2 may promote SBM and vascular channel formation primarily through Wnt/β‐catenin signaling. Additionally, osteoblastic ATP6AP2 may also promote the interaction and communication between osteoblasts, osteoclasts, and SBM cells, then enhancing the organization and remodeling of SBM. This function is evident in femur bone, as loss of ATP6AP2 in osteoblasts leads to reduced extracellular ATP and bisphosphate, and increased osteoclastic bone resorption [35, 36]. Thus, it is possible that loss of ATP6AP2 in osteoblasts disrupts Wnt/β‐catenin signaling cascade and v‐ATPase driven ATP release, leading to defective SBM formation, impaired channel connectivity to the meninges, and ultimately compromised SBM–meninges–cortex communication and impaired cognitive function. However, further studies are required to confirm this hypothesis.

In summary, our studies support a working hypothesis that AD‐linked mutations in osteoblasts may contribute to the development of AD or cognitive impairment by disrupting communication along the SBM–meninges–cortex axis. This axis may represent a critical pathway for early diagnosis and therapeutic intervention in AD.

## 4 Methods

### 4.1 Ethics Statement

All experimental procedures were approved by the Institutional Animal Care and Use Committee at Case Western Reserve University (IACUC, 2017‐0121 and 2017‐0115), according to US National Institutes of Health guidelines.

### 4.2 Animals and Regents

The mOCN‐Cre knock‐in mice were generated using the CRISPR‐Cas9 system by inserting a P2A‐iCre at the C‐terminus of the endogenous Bglap gene. The P2A peptide links iCre and Bglap, allowing independent expression of the iCre and Bglap proteins. The *hOcn‐Cre* transgenic mice were kindly provided by Dr. Tom Clemens (Johns Hopkins Medical School). The LSL‐APP_swe_ mice were generated using the pCCALL2 plasmid, as previously described [21]. The transcription of hAPP_swe_ [human APP695 transgene carrying the KM670/671NL (Swedish) mutations] in these mice is driven by the CAG promoter, while translation is prevented by a loxP–STOP–loxP (LSL) cassette. Consequently, hAPP_swe_ expression is regulated by both the CAG promoter and Cre‐mediated excision of the LSL sequence. Tg2576 mice, which express the hAPP_swe_ under the control of the hamster prion protein promoter [18], were obtained from Taconic (Hudson, NY, USA). The *APP^NL‐G‐F^* mice were kindly provided by Dr. Xin Qi (Case Western Reserve University); and the *Atp6ap2^flox/X^* mice were kindly provided by Dr. Frederique Yiannikouris (University of Kentucky, USA) and Dr. Genevieve Nguyen (INSERM, France). The Ai9 mice and LysM‐Cre mice were purchased from the Jackson Laboratory (Stock No 007909 and 004781). All mice were in C57BL/6J mouse background (for more than 6 generations).

The following antibodies were used, including Mouse monoclonal antibodies, such as hAPP (6E10, Cat#803001, BioLegend, San Diego, California, USA), β‐Catenin (610154, BD), β‐actin (A1978, Sigma Aldrich), Lrp4 (clone N207/27, University of California, Davis/National Institutes of Health NeuroMab Facility), RANK (Ab13918, Abcam) and GAPDH (#97166, Cell Signaling); Rabbit monoclonal antibodies, such as IBA1 (ab178846, Abcam), Runx2 (Ab192256, Abcam), Ki67 (Ab16667, Abcam) and NeuN (12943, Cell Signaling); Rabbit polyclonal antibodies ATP6AP2 (HPA003156, Sigma Aldrich) and CXCL12 (SDF1, PA5‐114344, Thermo Fisher Scientific); Goat polyclonal antibodies IBA1 (ab5076, Abcam) and CD206 (AF2535, R&D); Rat monoclonal antibodies, such as CD31 (65058‐1, Thermo Fisher Scientific), Endomucin (sc‐65495, Santa Cruz), Ly6c (ab54223, Abcam) and PODXL (MAB1556, R&D Systems); and Chicken polyclonal antibody GFAP (PA1‐10004, Thermo Fisher Scientific). Alexa Fluor 488 phalloidin (f‐actin, A12379) and Alexa Fluor 488 alpha‐smooth muscle actin (53‐9760‐82) were purchased from Thermo Fisher. Secondary antibodies were purchased from Jackson ImmunoResearch Laboratories, Inc. Other chemicals and reagents used in this study were of analytical grade.

ATP6AP2‐V5‐FL plasmid was purchased from DNASU (HsCD00446844). YFP‐APP_swe_ mutation (K670N/M671L, AAG ATG – AAC TTG) from the YFP‐APPWT construct by using the Q5 Site‐Directed Mutagenesis Kit (E0554S, New England Biolabs, Inc). The primers “CTGAAGTGAACTTGGATGCAGAATTCCGACATG” and “AGATCTCCTCCGTCTTGATATTTG” were used to generate the K670N/M671L mutation.

### 4.3 Primary OB‐Lineage Cell Cultures

Primary osteoblast cultures were isolated from mouse calvariae as described previously [57]. Briefly, calvariae were transferred to a dish containing PBS, and the soft tissues were carefully removed using tweezers. The cleaned bones were then cut into small fragments of approximately 1–2 mm^2^ and sequentially digested with collagenase solution (37°C, 30 min, twice) and trypsin solution (37°C, 30 min) to remove residual soft tissue and adherent cells. After an additional collagenase digestion, the bone fragments were washed three times with culture medium and transferred to 60 mm culture dishes containing DMEM supplemented with 10% FBS, 1% penicillin/streptomycin, 10 mmol/L β‐glycerophosphate, and 50 µmol/L L‐ascorbic acid‐2‐phosphate. The culture medium was replaced three times per week. Osteoblasts began to migrate from the bone chips after 3–5 days. After two weeks, the monolayer cells were detached by incubation with trypsin solution and replated onto 100 mm tissue culture dishes in α‐MEM supplemented with 10% FBS and 1% penicillin/streptomycin.

### 4.4 Cell Lysis and Western Blot

Cells were lysed in lysis buffer containing 50 mmol/L Tris‐HCl (pH 7.5), 150 mmol/L NaCl, 1%(v/v) Triton X‐100, 0.1% SDS, 0.5% deoxycholate, and 1 mmol/L EDTA, supplemented with protease inhibitors (1 µg/mL leupeptin and pepstatin, 2 µg/mL aprotinin, and 1 mmol/L PMSF) and phosphatase inhibitors (10 mmol/L NaF and 1 mmol/L Na_3_VO_4_). Whole cell extracts were fractionated by SDS‐PAGE and transferred to a nitrocellulose membrane (Bio‐Rad). After incubation with 5% BSA in TBST (10 mmol/L Tris, 150 mmol/L NaCl, 0.5% Tween 20, pH 8.0) for 1‐h, the membrane was incubated with indicated antibodies overnight at 4 °C. Membranes were washed with TBST for three times and incubated with a 1:5 000 dilution of horseradish peroxidase‐conjugated anti‐mouse or anti‐rabbit antibodies for 1‐h. Blots were washed with TBST three times and developed with the ECL system (Bio‐Rad).

### 4.5 Immunofluorescence Staining and Imaging Analysis

Mice were transcardially perfused with PBS followed by 4% (w/v) paraformaldehyde (PFA) in phosphate‐buffered saline (PBS, pH 7.4) to remove intravascular plasma proteins. The brain and bones were then extracted and post‐fixed in 1% PFA at 4 °C overnight. Brain tissues were sectioned into 40 µm‐thick transverse slices using a vibratome (Leica VT1000 S) and stored in FD Section Storage Solution. Bone samples were decalcified in EDTA for one week, embedded in OCT compound, and sectioned into 40 µm slices using a freezing microtome. The sections were washed with PBS and blocked in PBS containing 0.5% Triton X‐100 and 10% donkey serum for 1 h at room temperature, followed by incubation with primary antibodies overnight at 4 °C. After three PBS washes, sections were incubated with the appropriate fluorophore‐conjugated secondary antibodies for 1 h at room temperature. Nuclei were counterstained with DAPI. Stained sections were imaged at room temperature using a confocal microscope, and fluorescence quantification was performed with ZEN software according to the manufacturer's instructions (Carl Zeiss).

### 4.6 RNA Isolation and Real Time‐PCR

Total RNA was isolated by Trizol extraction (Invitrogen, Carlsbad, CA, USA). Q‐PCR was performed by using Quantitect SYBR Green PCR Kit (Bio‐Rad) with a Real‐Time PCR System (Opticon Monitor 3). Bglap primers (5ʹ‐GAGGGCAATAAGGTAGTGAA‐3ʹ and 5ʹ‐CATAGATGCGTTTGTAGGC‐3ʹ), iCre primers (5ʹ‐TTTGAACGCACTGACTTTG‐3ʹ and 5ʹ‐TCAGCATTCTCCCACCAT‐3ʹ) and β‐actin (5ʹ‐AGGTCATCACTATTGGCAACGA‐3ʹ and 5ʹ‐CATGGATGCCACAGGATTCC‐3ʹ) were used.

### 4.7 Micro‐Computed Tomography (µCT)

The µCT analyses were conducted as previously described [35, 36, 58]. Briefly, mouse skull bone were collected, fixed in 10% formaldehyde for 24 h, and then were scanned using the Siemens Inveon Micro PET/CT scanner (10‐µm resolution) or Mediso nanoScan SPECT/CT/PET system. The linear attenuation is converted to mineral density based on a hydroxyapatite calibration standard. Scans were automatically reconstructed into 2D slices, and the 3‐D reconstruction was performed using all the outlined slices (Amira 3D). The regions of interest in the midline from the parietal bones were identified and analyzed for each individual mouse. Data were obtained on skull bone thickness, skull bone marrow area, skull bone marrow density, and channel density.

### 4.8 Cerebral Cortical Blood Flow Measurements

Cerebral cortical blood flow was measured using laser speckle contrast imaging (LSCI). Mice were anesthetized with isoflurane and secured in a digital stereotaxic apparatus. The operator was blinded to group allocation. The scalp and underlying connective tissues were carefully opened to fully expose the skull surface. The LSCI system was focused on the exposed skull to obtain a clear speckle contrast image. Normal saline was applied periodically to maintain skull moisture during imaging. Cortical blood flow from both hemispheres was recorded for 2 min each, and the average perfusion units (PU) over the recording period were quantified and compared between groups.

### 4.9 Behavioral Tests

Male mice aged 6 or 12 months were subjected to behavioral assessments. All behavioral experiments were conducted by investigators blinded to genotype. Prior to testing, mice were transferred to the behavioral testing room and acclimated for at least 4 h. All testing apparatuses were cleaned with 70% ethanol between trials to eliminate olfactory cues.

The Open Field Test (OFT) was performed as previously described [59]. Briefly, each mouse was placed in an open‐field chamber (50 × 50 × 20 cm, L × W × H) under a light intensity of approximately 150 lux. Animal movement was recorded for 10 min using an overhead video camera and analyzed with EthoVision tracking software (Noldus). Total distance traveled and time spent in the center zone (25 × 25 cm) were quantified.

The Morris Water Maze (MWM) test was conducted as described previously [60]. The apparatus consisted of a circular pool (120 cm in diameter) filled with water made opaque by the addition of a nontoxic white coloring agent (Soft Gel Paste Food Color, AmeriColor). A hidden escape platform (10 cm in diameter) was submerged 1 cm below the water surface. Mice were trained over five consecutive days, with four trials per day (60 s per trial, 20 min inter‐trial interval) to locate the hidden platform using eight distinct spatial cues placed around the pool. On day 6, a probe trial was performed in which the platform was removed and mice were released from a novel starting location. The time spent in each quadrant, the number of platform crossings, and swim speed were analyzed using EthoVision (Noldus). All analyses were conducted by experimenters blinded to genotypes.

The Novel Object Recognition (NOR) task was performed as described previously [61], consisting of a habituation phase followed by a testing phase. During habituation, each mouse freely explored an empty arena for 5 min per day over two consecutive days. On the third day, the testing phase began. During the familiarization trial, mice were placed in the arena containing two identical objects, released at the center of the opposite wall with their back to the objects, and allowed to explore freely for 5 min. Object interaction was defined as nose or whisker contact, or close investigation within the object zone. After a 4‐h retention interval, the test trial was performed with one familiar object and one novel object for 5 min. To measure of an animal's preference for a novel object over a familiar one, the discrimination index (DI) was calculated by dividing the difference in exploration time between the novel and familiar objects by the total exploration time.

The Y‐maze spontaneous alternation task was performed as previously described [62]. Each mouse was placed at the center of the three opaque arms and allowed to freely explore for 8 min. The total number of arm entries and the percentage of spontaneous alternations were recorded and analyzed.

### 4.10 Bulk RNA‐Sequencing

Total RNA was extracted from purified Td^+^ osteoblasts isolated by flow cytometry from *mOCN‐Cre; Ai9* and *TgAPP_swe_^mOCN‐Cre^; Ai9* mice. RNA integrity was assessed for each sample, and only samples with a RNA Integrity Number (RIN) ≥ 7 were included for further analysis. RNA‐seq was performed by BGI America (Cambridge, MA) using the DNBseq platform. Gene expression levels were normalized as fragments per kilobase of transcript per million mapped reads (FPKM). Differentially expressed genes (DEGs) were identified using DESeq2 and PoissonDis algorithms, with *P*‐values adjusted by the Benjamini–Hochberg (BH) method. Genes with adjusted *P* ≤ 0.05 and |log_2_ fold change| ≥ 1 were considered significantly differentially expressed. Heatmaps were generated using the online data analysis and visualization platform https://www.bioinformatics.com.cn, with gene expression values Z‐score transformed. The list of secreted proteins was obtained from the UniProt database (https://www.uniprot.org). Gene Ontology (GO) enrichment analysis was performed using Metascape (https://metascape.org), and GO terms with *P* ≤ 0.05 were considered significantly enriched.

### 4.11 Bone Marrow Macrophage Transplantation

Bone marrow macrophage transplantation was performed as previously described [63, 64]. Briefly, recipient mice were conditioned with busulfan prior to transplantation. Mice were housed in sterile cages with autoclaved bedding, food, and water for one week before the initiation of busulfan treatment. Freshly prepared sterile busulfan was administered intraperitoneally at a dose of 20 mg/kg once daily for two consecutive days. Twenty‐four hours after the final dose, bone marrow cells were harvested from the long bones of sex‐matched donor mice, resuspended in ice‐cold PBS, and filtered through a 70 µm cell strainer. A total of 5 × 10^6^ donor bone marrow cells in 0.2 mL PBS were injected into each recipient mouse via the tail vein. Following transplantation, mice were maintained under sterile conditions with sterilized bedding, food, and water for at least 3 weeks.

### 4.12 Statistical Analysis

All experimental quantifications, including behavioral tests, histological analyses, immunofluorescence image quantifications, and other image‐based measurements, were performed by investigators blinded to the genotype and treatment groups to minimize bias. For in vivo studies, 6–10 mice per genotype per assay were used. For in vitro cell biological and biochemical studies, each experiment was repeated 3 times. Data were analyzed by Student *t*‐test, two‐way ANOVA and post‐hoc test (GraphPad Prism 8). The significance level was set at P < 0.05 (*P < 0.05, **P < 0.01, ***P < 0.001).

## Author Contributions

L.X. and W‐C.X designed the project and wrote the manuscript. L.X. performed most experiments. D.S. helped to design and create the mOCN‐Cre knock‐in mice. H.G. helped to collect some brain and bone samples. D.L. and Z.L help to perform behavioral tests. L.M. and W.‐C.X. helped data analysis, interpretation, and supervised the project, and W.‐C. X. obtained the funding support for the project.

## Conflicts of Interest

The authors declare no conflicts of interest.

## Supporting information




**Supporting File**: advs75622‐sup‐0001‐SuppMat.docx.

## Data Availability

RNA‐seq data generated in this study have been deposited in the NCBI Gene Expression Omnibus (GEO) under accession number GSE310893 and are publicly available. All other data supporting the findings of this study are available from the corresponding author upon reasonable request.
