# Supplementary material for: A Skull Bone Marrow‐to‐Brain Axis Links Osteoblastic Activity to Myeloid Cell Trafficking, Cerebral Blood Flow, and Cognition in Alzheimer's Progression
Source: Adv Sci (Weinh). 2026 May 10;13(43):e75622. doi: 10.1002/advs.75622 (PMC13336015; doi:10.1002/advs.75622)
Supplement: Supplementary file 1 — Supporting File: advs75622‐sup‐0001‐SuppMat.docx. [file ADVS-13-e75622-s001.docx]

**Supplementary information**

**A skull bone marrow-to-brain axis links osteoblastic activity to myeloid cell trafficking, cerebral blood flow, and cognition in Alzheimer’s progression**

Lei Xiong^1^, Dong Sun^1^, Hao-Han Guo^1^, Daehoon Lee^1^, Zhipeng Liu^1^, Lin Mei^1,2^, and Wen-Cheng Xiong^1*^

^1^, Department of Neurosciences, School of Medicine, Case Western Reserve University, Cleveland, OH, USA

^2^, Chinese Institute of Medical Research, Capital Medical University, Beijing, China

*Corresponding author

Email: [Wen-Cheng.Xiong@case.edu](mailto:Wen-Cheng.Xiong@case.edu)


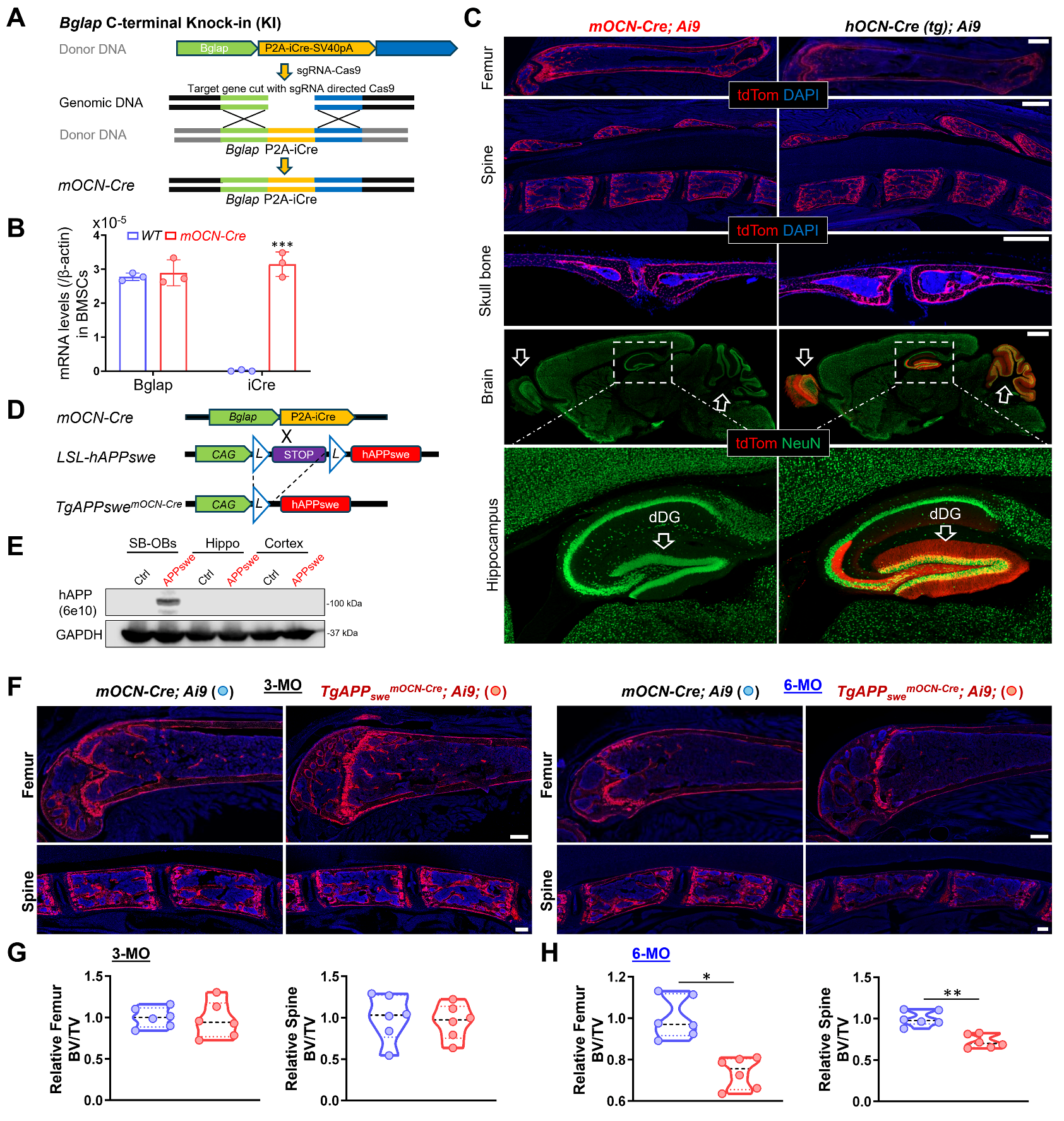
**Figure S1**

**Figure S1. Generation of *mOCN-Cre* knock-in (ki) and *TgAPP_swe_ ^mOCN-Cre^* mice.**

**A**, Illustration of generation of *mOCN-Cre* knock-in mice. iCre was inserted into the C-terminus of endogenous Bglag, and P2A peptide was used to link iCre and Bglap.

**B**, RT-PCR analysis of Bglap and iCre expression in BMSCs of 3-MO *WT* and *mOCN-Cre* mice.

**C**, Representative images of femur, spine, skull bone and brain sections from 3-MO *mOCN-Cre; Ai9* and *hOcn-Cre;Ai9* mice coimmunostained with DAPI (blue) or NeuN (green). Cre-positive cells express the red fluorescent protein tdTomato (red). Scale bar, 1 mm.

**D**, Illustration of generation of the conditional transgenic mice selectively expressing human APP_swe_ in an mOCN-Cre dependent manner.

**E**, Western blotting analysis of hAPP (6e10) expression in primary cultured skull OBs, hippocampal and cortical tissue from 3-MO male *Ctrl* and *TgAPP_swe_^mOCN-Cre^* mice. GAPDH was used as the loading controls.

**F**, Representative images of femur and spine sections from 3-MO or 6-MO *mOCN-Cre; Ai9* and *TgAPP_swe_^mOCN-Cre^; Ai9* mice immunostained with DAPI (blue). Cre-positive cells express the red fluorescent protein tdTomato (red). Scale bar, 0.5 mm.

**G-H**, Quantification analyses of femur and spine bone volumes over total volumes (BV/TV) in F.

Data in (**B**) are presented as mean ± SD (n = 3). Data in (**G**) and (**H**) are shown as violin plots together with individual data points, and dot lines and dash line represent the quartiles and the median, respectively (n = 6 male mice of each genotype). P values obtained by unpaired two-tailed t test. *P < 0.05. **P < 0.01. ***P < 0.001.


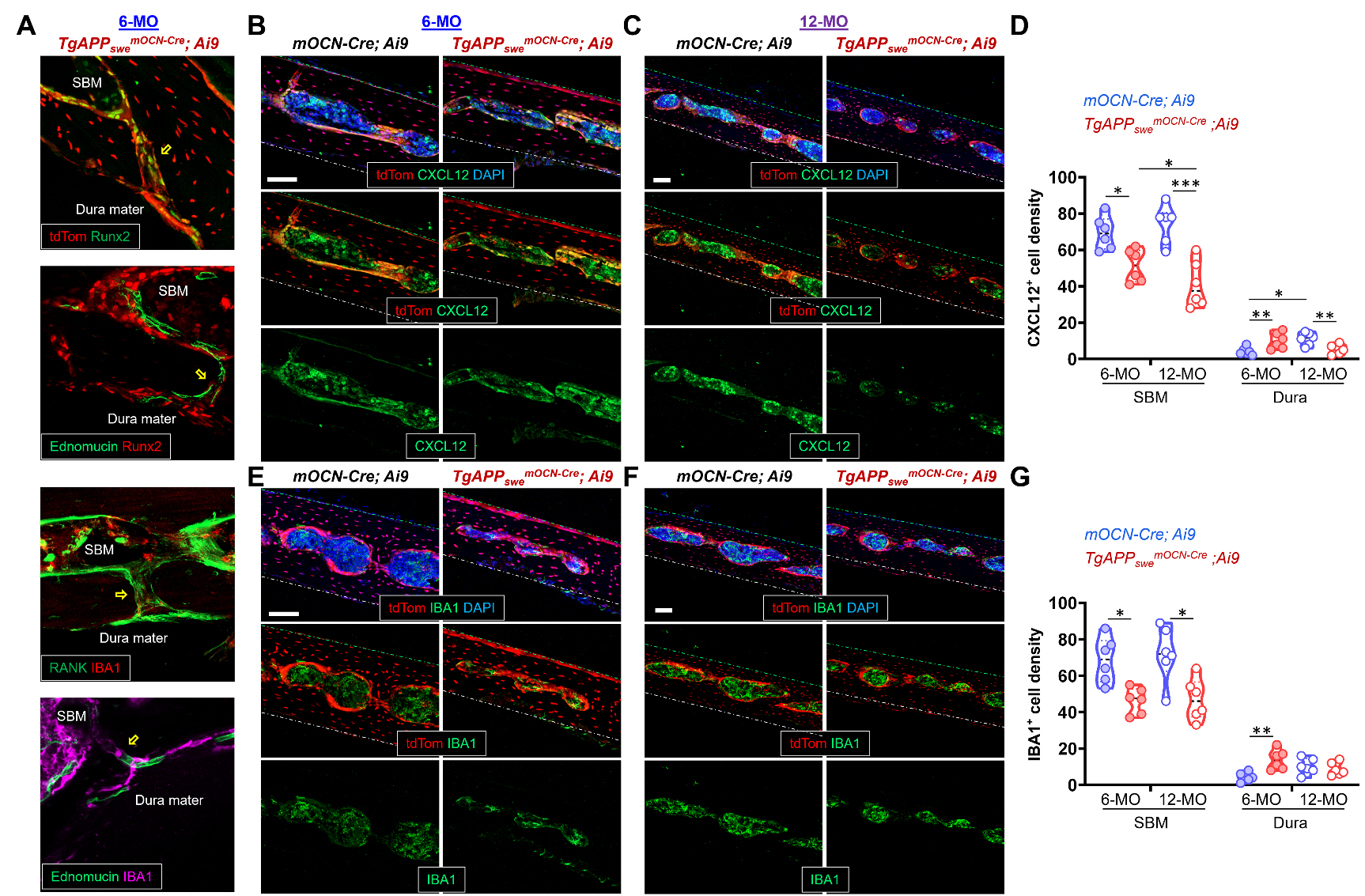
**Figure S2**

**Figure S2. Increased dura mater side myeloid cells distribution in 6-MO *TgAPP_swe_^mOCN-Cre^* mice.**

**A**, Representative channel images of skull bone sections from 6-MO male *TgAPP_swe_^mOCN-Cre^; Ai9* mice coimmunostained with Runx2, Ednomucin, RANK and IBA1.

**B-C**, Representative images of skull bone sections from 6-MO (**B**) and 12-MO (**C**) male *mOCN-Cre; Ai9* and *TgAPP_swe_^mOCN-Cre^; Ai9* mice coimmunostained with DAPI (blue) and CXCL12 (green, B). Cre-positive cells express the tdTomato (red). Scale bar, 100 μm.

**D**, Quantification analyses of CXCL12^+^ cell density in SBM and dura side in B and C.

**E-F**, Representative images of skull bone sections from 6-MO (**E**) and 12-MO (**F**) male *mOCN-Cre; Ai9* and *TgAPP_swe_^mOCN-Cre^*; Ai9 mice coimmunostained with DAPI (blue) and IBA1 (green, H). Cre-positive cells express the tdTomato (red). Scale bar, 100 μm.

**G**, Quantification analyses of IBA1^+^ cell density in SBM and dura side in E and F.

Data in (**D**) and (**G**) are shown as violin plots together with individual data points, and dot lines and dash line represent the quartiles and the median, respectively (n = 6). P values obtained by two-way ANOVA followed by Bonferroni’s post hoc test. *P < 0.05. **P < 0.01. ***P < 0.001.


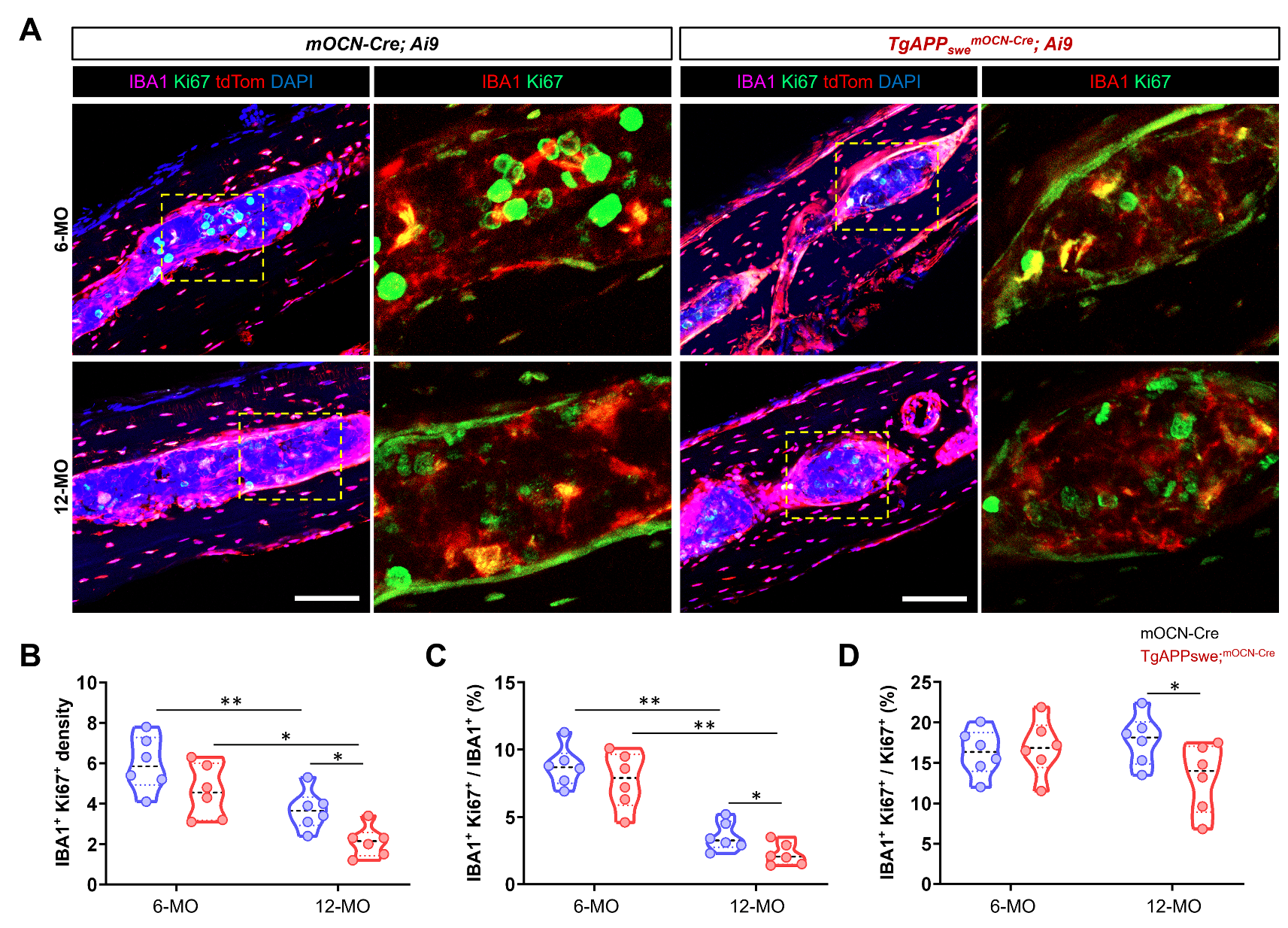
**Figure S3**

**Figure S3. Age-dependent decline in proliferation of SBM IBA1⁺ macrophages in *TgAPP_swe_^mOCN-Cre^* mice.**

**A**, Representative images of skull bone sections from 6-MO and 12-MO *mOCN-Cre; Ai9* and *TgAPP_swe_^mOCN-Cre^; Ai9* mice coimmunostained with IBA1, Ki67 and DAPI. Cre-positive cells express the tdTomato (red). Scale bar, 100 μm.

**B-D**, Quantification analyses of IBA1^+^ Ki67^+^ cell density and percentage of IBA1^+^ Ki67^+^ cells among IBA1^+^ or Ki67^+^ cells in A.

Data in (**B-D**) are shown as violin plots together with individual data points, and dot lines and dash line represent the quartiles and the median, respectively (n = 6 male mice of each genotype). Statistical analysis was performed using two-way ANOVA followed by Bonferroni’s post hoc test. *P < 0.05. **P < 0.01. ***P < 0.001.


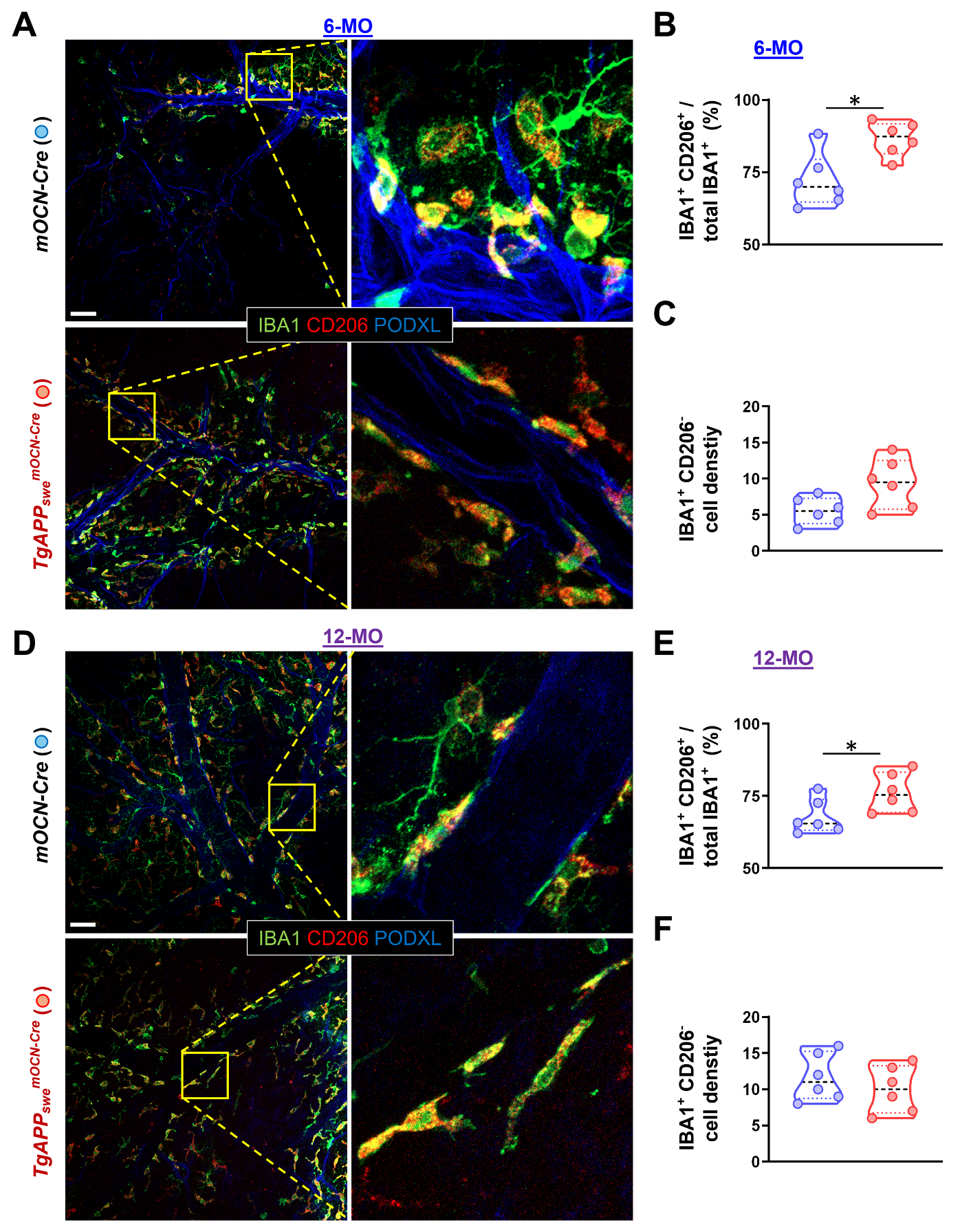
**Figure S4**

**Figure S4. Increased macrophage (CD206^+^) cell number in the meninges of 6-MO *TgAPPswe^Ocn-Cre (ki)^;Ai9* mice.**

**A**, Representative images of cortical surface sections from 6-MO *mOCN-Cre; Ai9* and *TgAPP_swe_^mOCN-Cre^; Ai9* mice coimmunostained with IBA1, CD206 and PODXL. Scale bar, 50 μm.

**B-C**, Quantification analyses of percentage of CD206^+^ IBA1^+^ cells and IBA1^+^ CD206^-^ cell density in A.

**D**, Representative images of cortical surface sections from 12-MO *mOCN-Cre; Ai9* and *TgAPP_swe_^mOCN-Cre^; Ai9* mice coimmunostained with IBA1 and PODXL or SMA. Scale bar, 50 μm.

**E-F**, Quantification analyses in D.

Data in (**B-C**) and (**E-F**) are shown as violin plots together with individual data points, and dot lines and dash line represent the quartiles and the median, respectively. n = 6 male mice of each genotype. P values obtained by unpaired two-tailed t test. *P < 0.05.


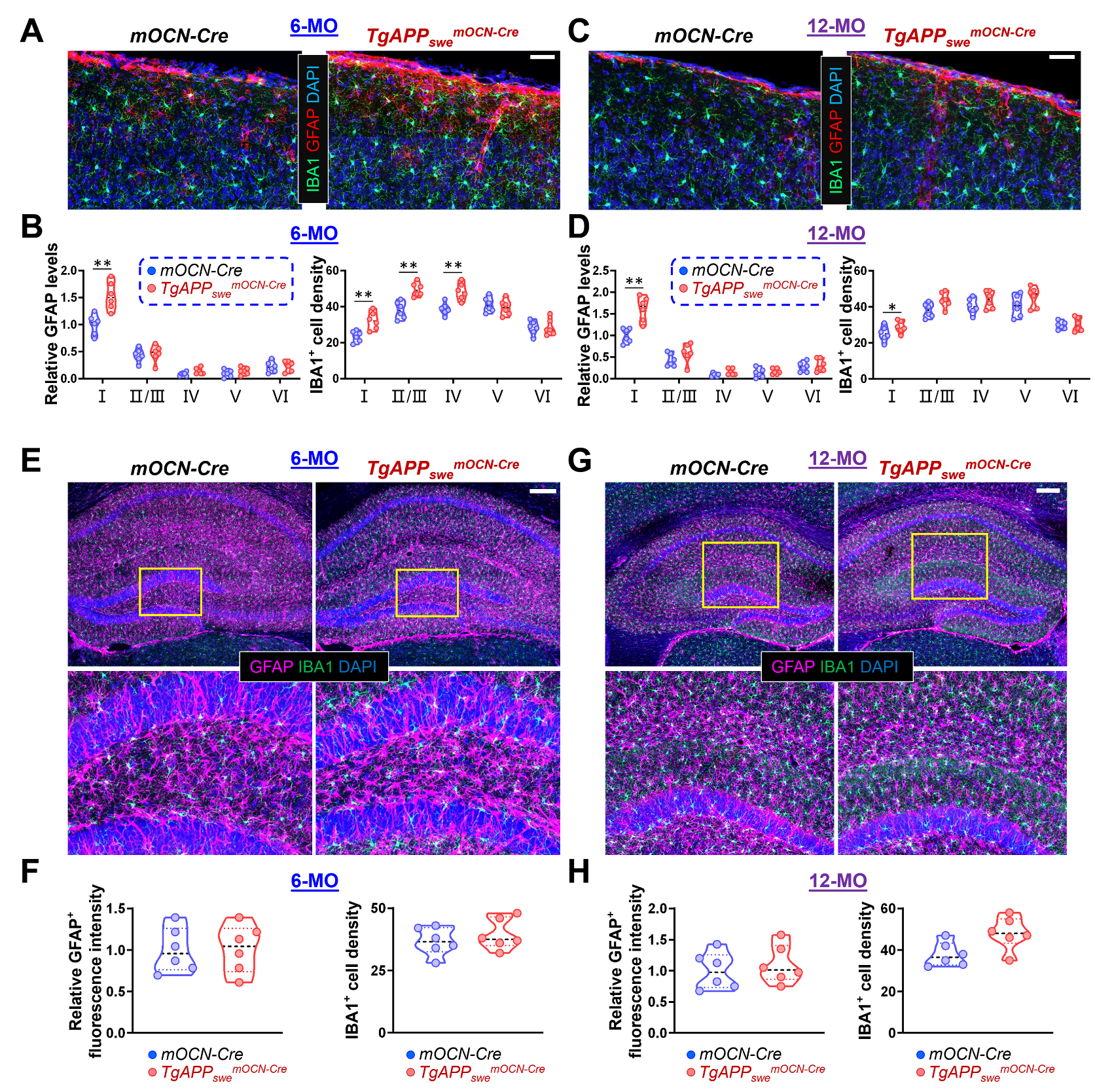
**Figure S5**

**Figure S5. Increased astrocytes (GFAP^+^) and microglia/macrophage (IBA1^+^) cell number in the cortex region of 6-MO *TgAPP_swe_^mOCN-Cre^* mice.**

**A-D**, Representative images of co-immunostaining with IBA1 (green), GFAP (red), and DAPI (blue) of cortex sections from 6-MO (A) or 12-MO (C) *mOCN-Cre; Ai9* and *TgAPP_swe_^mOCN-Cre^*; Ai9 mice. Scale bar, 50 μm. Quantification analyses of GFAP levels and IBA1^+^ cell density are presented in B and D.

**E-H**, Representative images of co-immunostaining with IBA1 (green), GFAP (magenta), and DAPI (blue) of hippocampal sections from 6-MO (E) or 12-MO (G) *mOCN-Cre; Ai9* and *TgAPP_swe_^mOCN-Cre^*; Ai9 mice. Scale bar, 200 μm. Quantification analyses of GFAP levels and IBA1^+^ cell density are presented in F and H.

Data in (**B**), (**D**), (**F**) and (**H**) are shown as violin plots together with individual data points, and dot lines and dash line represent the quartiles and the median, respectively (n = 6 male mice of each genotype). P values obtained by unpaired two-tailed t test. **P < 0.01.


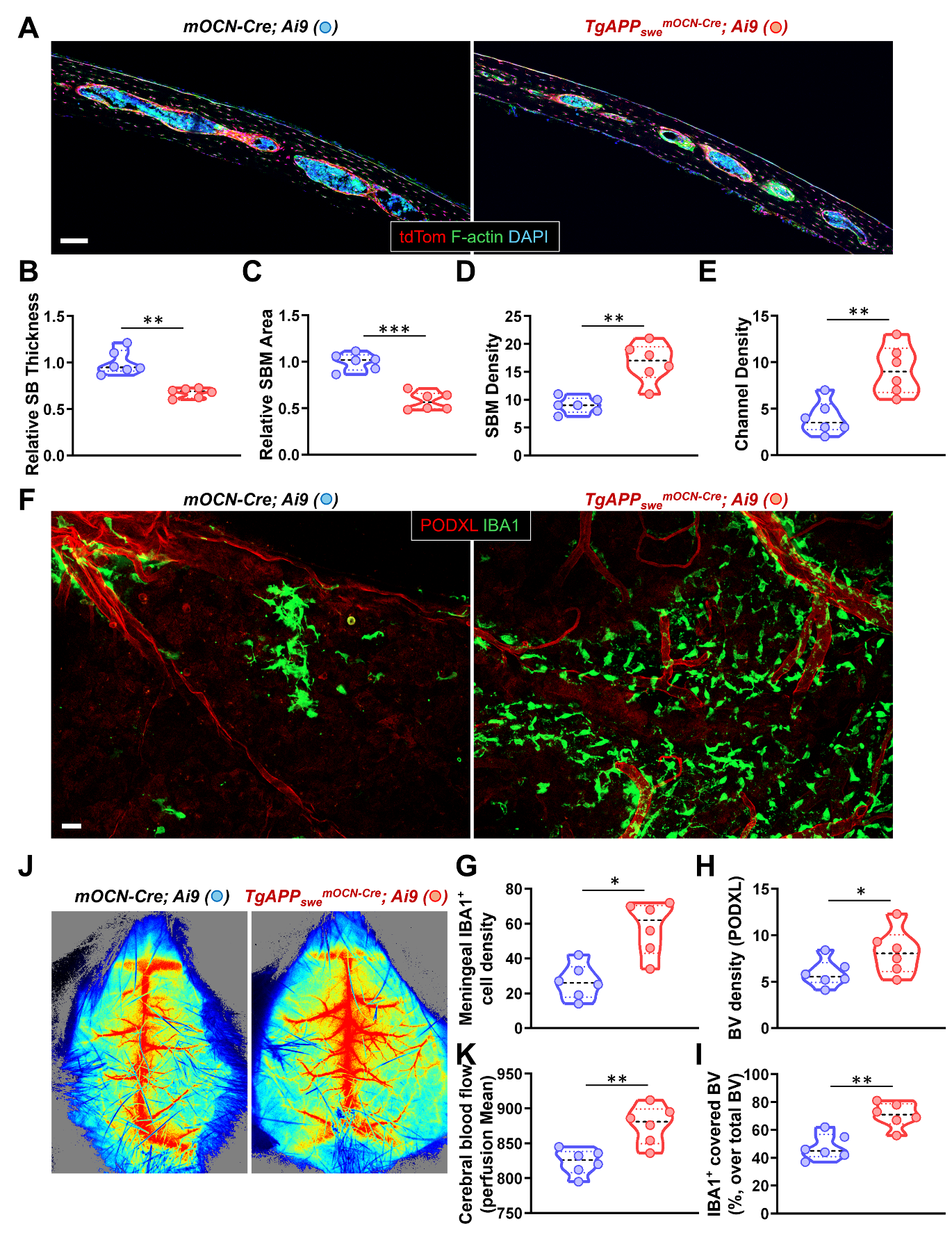
**Figure S6**

**Figure S6. Slimier phenotype in 6-MO female TgAPP_swe_^mOCN-Cr^ ;Ai9 mice.**

**A**, Representative images of skull bone sections from 6-MO female *mOCN-Cre; Ai9* and *TgAPP_swe_^mOCN-Cre^; Ai9* mice coimmunostained with f-actin (green) and DAPI (blue). Cre-positive cells express the tdTomato (red). Scale bar, 100 μm.

**B-E**, Quantification analyses of relative SB thickness, SBM area, SBM density and channel density in A.

**F**, Representative images of cortical surface sections from 6-MO female *mOCN-Cre; Ai9* and *TgAPP_swe_^mOCN-Cre^; Ai9* mice coimmunostained with IBA1 and PODXL. Scale bar, 40 μm.

**G-I**, Quantification analyses of meninges IBA1^+^ cell density, blood vessels (BV) density, and percentage of IBA1^+^ cells covered BV in F.

**J**, Representative image of unilateral cerebral blood flow analyses of 6-MO female *mOCN-Cre* and *TgAPP_swe_^mOCN-Cre^* mice via laser speckle contrast imaging.

**K**, Quantification of average recorded perfusion in J.

Data in (**B-E**), **(G-I)** and (**K**) are shown as violin plots together with individual data points, and dot lines and dash line represent the quartiles and the median, respectively (n = 6 female mice of each genotype). P values obtained by unpaired two-tailed t test. *P < 0.05. **P < 0.01. ***P < 0.001.


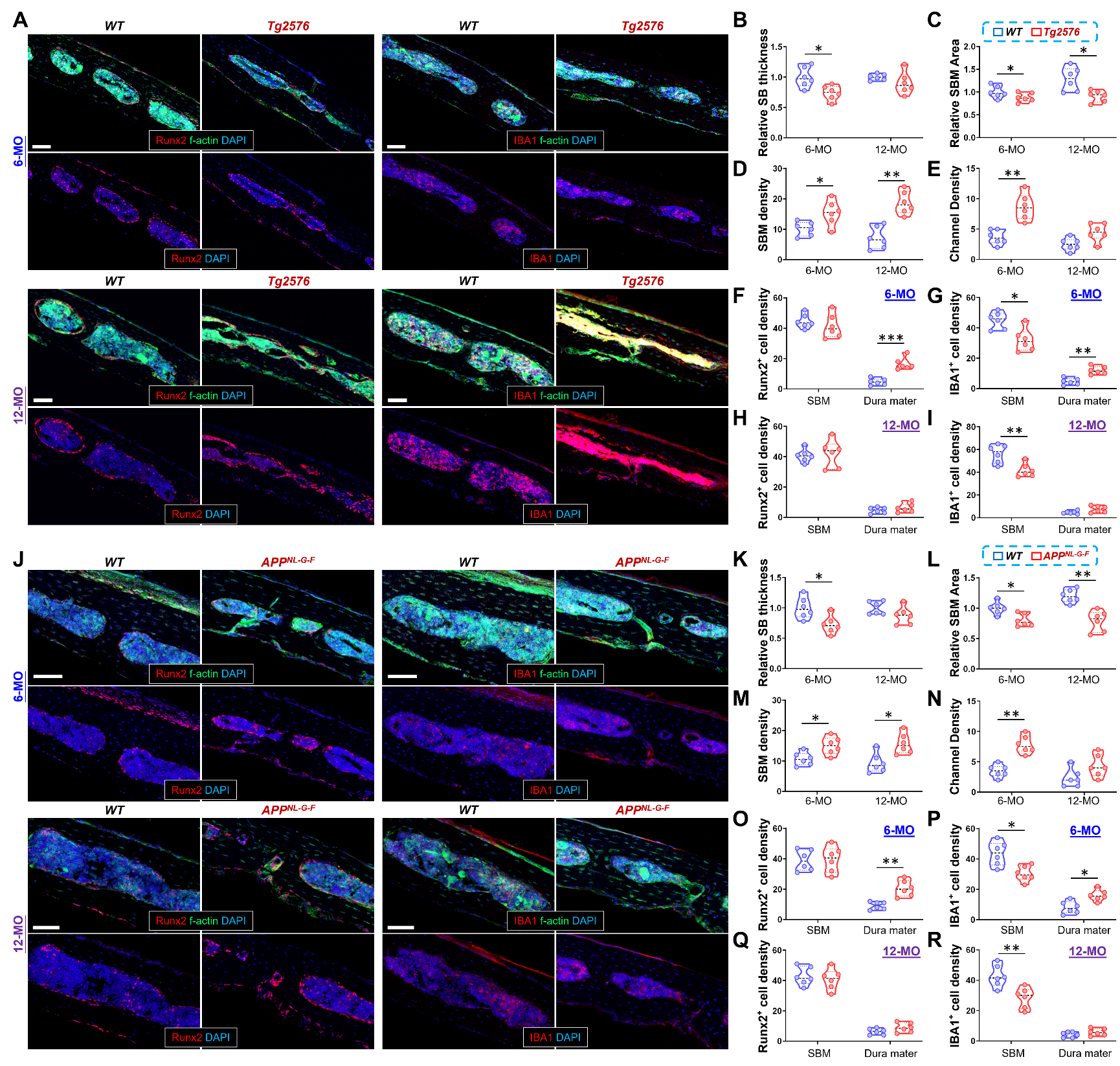
**Figure S7**

**Figure S7. Altered skull bone remodeling in *Tg2576* mice and *APP^NL-G-F^* mice.**

**A**, Representative images of skull bone sections from 6-MO or 12-MO *WT* and *Tg2576* mice coimmunostained with Runx2, f-actin, IBA1 and DAPI. Scale bar, 100 μm.

**B-I**, Quantification analyses in A.

**J**, Representative images of skull bone sections from 6-MO or 12-MO *WT* and *APP^NL-G-F^* mice coimmunostained with Runx2, f-actin, IBA1 and DAPI. Scale bar, 100 μm.

**K-R**, Quantification analyses in J.

Data in (**B-I**) and (**K-R**) are shown as violin plots together with individual data points, and dot lines and dash line represent the quartiles and the median, respectively (n = 6). P values obtained by unpaired two-tailed t test. *P < 0.05. **P < 0.01. ***P < 0.001.


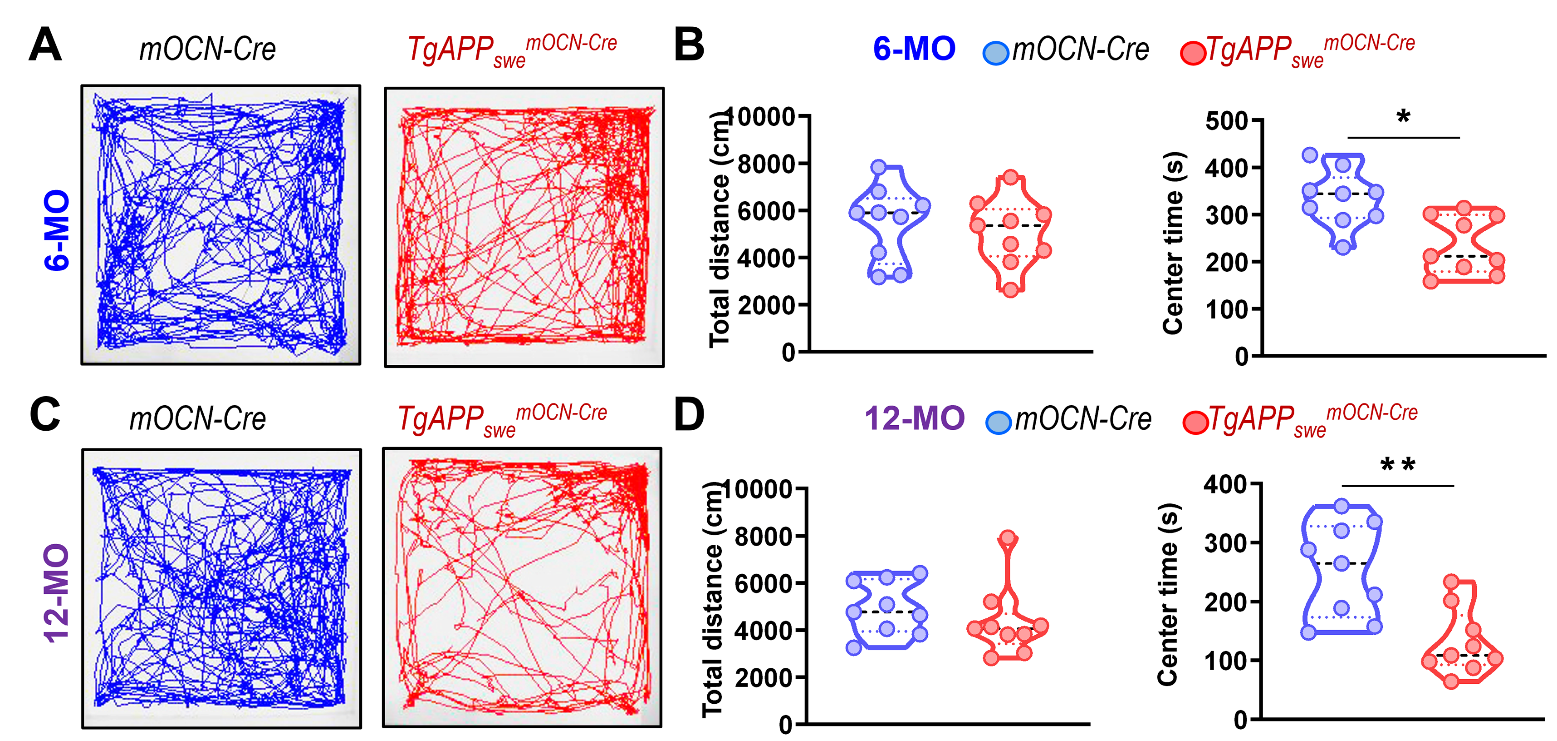
**Figure S8**

**Figure S8. Promoting age-dependent anxiety by Tg*APP_swe_^mOCN-Cre^*.**

**A-D**, Open field test (OFT) of 6-MO and 12-MO *mOCN-Cre* and *TgAPP_swe_^mOCN-Cre^* male mice. Representative tracing images were shown in a and c. Quantifications of total distance and center duration time were shown in B and D.

Data in (**B**) and (**D**) are shown as violin plots together with individual data points, and dot lines and dash line represent the quartiles and the median, respectively. (n=9 animals per genotype). P values obtained by unpaired two-tailed t test. *P < 0.05. **P < 0.01.


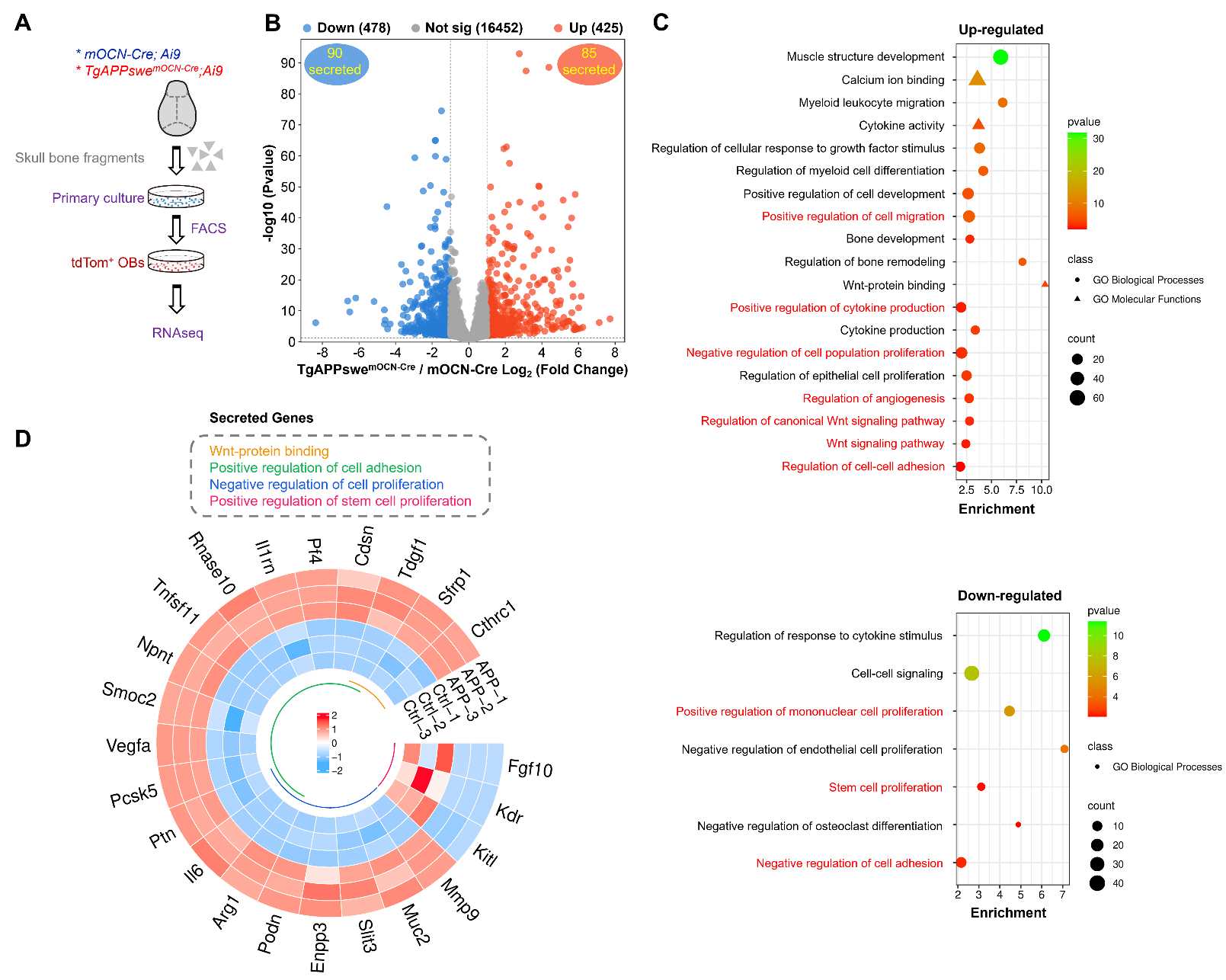
**Figure S9**

**Figure S9. Changes in multi-pathways, including down-regulation of cell proliferation, in APP_swe_^+^ calvarial osteoblasts.**

**A**, Schematic of purification and RNA-seq of Tdtomato^+^ OBs from skull bone of 6-MO *mOCN-Cre; Ai9* and *TgAPP_swe_^mOCN-Cre^;Ai9* male mice.

**B**, Volcano plots.

**C**, GO analysis of up/down-regulated genes.

**D**, Heat map of differentially expressed secreted genes identified by RNA-seq.


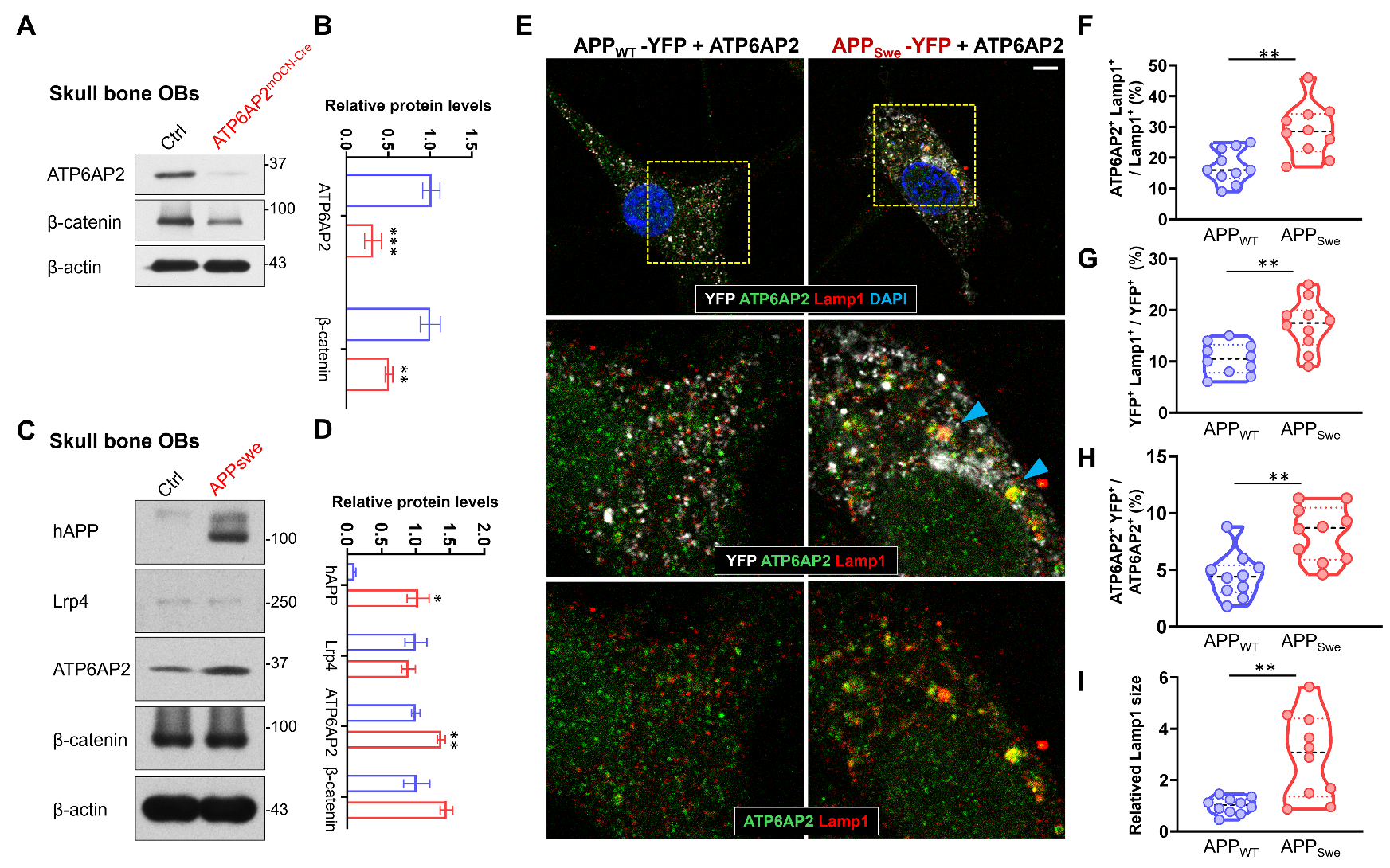
**Figure S10**

**Figure 10. Differential regulation of ATP6AP2 expression and lysosomal localization in APP_swe_-expressing osteoblasts.**

**A-B**, Decreased beta-catenin expression in skull bone OBs derived from 3-MO *ATP6AP2^mOCN-Cre^* mice by Western blots. Quantification analysis was shown in B.

**C-D**, Increased ATP6AP2 expression in skull bone OBs derived from 3-MO *TgAPP_swe_^mOCN-Cre^* mice by Western blots. Quantification analysis was shown in D.

**E,** Immunostaining analysis of ATP6AP2 and Lamp1 in MC3T3 cells transfected with the APP_WT_-YFP or APP_swe_-YFP. Images marked with yellow squares were amplified and shown below. Arrows indicate enlarged lysosomes with colocalization of ATP6AP2 and YFP. Bar, 10 µm.

**F-I,** Quantification analyses of E.

Data in (**B**) and (**D**) are presented as mean ± SD (n = 3). Data in (**F-I**) are shown as violin plots together with individual data points, and dot lines and dash line represent the quartiles and the median, respectively (n = 10 cells). P values obtained by unpaired two-tailed t test. *P < 0.05. **P < 0.01. ***P < 0.001.


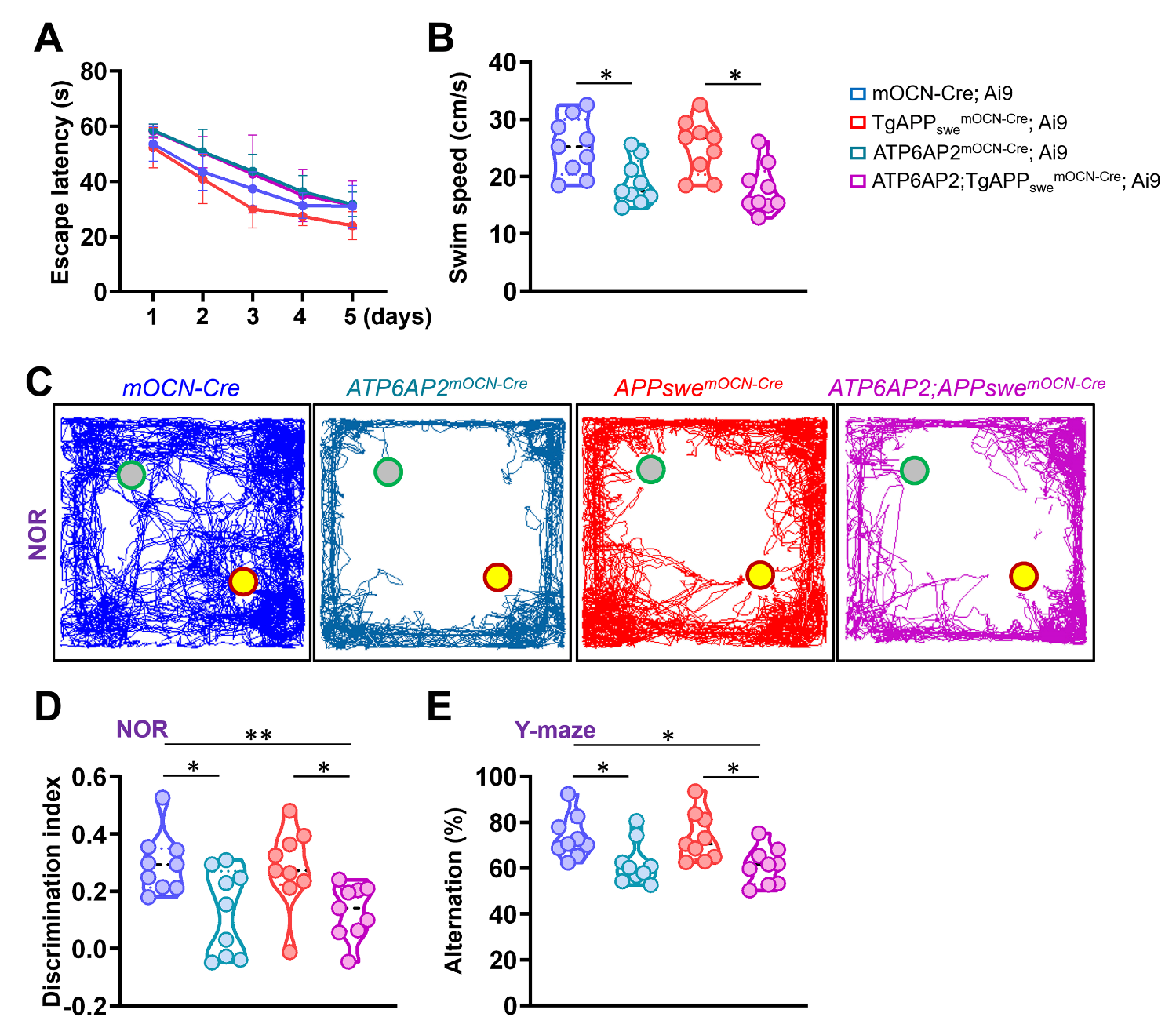
**Figure S11**

**Figure S11. Cognition decline in *ATP6AP2;TgAPP_swe_^mOCN-Cre^* mice**

**A-B**, MWM. The latencies to reach the hidden platform during the training period were showed in A, and swim speed was shown in B.

**C-D**, Novel Object Recognition (NOR). The discrimination index was shown in D.

**E**, Y-maze test. Spontaneous alternation was shown.

Data in (**A**) is presented as mean± SD (n=9). Data in (**B**), (**D**) and (**E**) are shown as violin plots together with individual data points, and dot lines and dash line represent the quartiles and the median, respectively. (n=9 animals per genotype). Statistical analysis was performed using one-way ANOVA followed by Tukey’s post hoc test. *P < 0.05. **P < 0.01.
